# Synthetic viability genomic screening defines Sae2 function in DNA repair

**DOI:** 10.15252/embj.201590973

**Published:** 2015-04-21

**Authors:** Fabio Puddu, Tobias Oelschlaegel, Ilaria Guerini, Nicola J Geisler, Hengyao Niu, Mareike Herzog, Israel Salguero, Bernardo Ochoa-Montaño, Emmanuelle Viré, Patrick Sung, David J Adams, Thomas M Keane, Stephen P Jackson

**Affiliations:** 1 The Gurdon Institute and Department of Biochemistry, University of CambridgeCambridge, UK; 2 The Wellcome Trust Sanger InstituteHinxton, Cambridge, UK; 3 Molecular Biophysics and Biochemistry, Yale University School of MedicineNew Haven, CT, USA

**Keywords:** Mre11, Sae2, suppressor screening, synthetic viability, whole-genome sequencing

## Abstract

DNA double-strand break (DSB) repair by homologous recombination (HR) requires 3′ single-stranded DNA (ssDNA) generation by 5′ DNA-end resection. During meiosis, yeast Sae2 cooperates with the nuclease Mre11 to remove covalently bound Spo11 from DSB termini, allowing resection and HR to ensue. Mitotic roles of Sae2 and Mre11 nuclease have remained enigmatic, however, since cells lacking these display modest resection defects but marked DNA damage hypersensitivities. By combining classic genetic suppressor screening with high-throughput DNA sequencing, we identify Mre11 mutations that strongly suppress DNA damage sensitivities of *sae2*Δ cells. By assessing the impacts of these mutations at the cellular, biochemical and structural levels, we propose that, in addition to promoting resection, a crucial role for Sae2 and Mre11 nuclease activity in mitotic DSB repair is to facilitate the removal of Mre11 from ssDNA associated with DSB ends. Thus, without Sae2 or Mre11 nuclease activity, Mre11 bound to partly processed DSBs impairs strand invasion and HR.

## Introduction

The DSB is the most cytotoxic form of DNA damage, with ineffective DSB repair leading to mutations, chromosomal rearrangements and genome instability that can yield cancer, neurodegenerative disease, immunodeficiency and/or infertility (Jackson & Bartek, 2009). DSBs arise from ionising radiation and radiomimetic drugs and are generated when replication forks encounter single-stranded DNA breaks or other DNA lesions, including DNA alkylation adducts and sites of abortive topoisomerase activity. DSBs are also physiological intermediates in meiotic recombination, being introduced during meiotic prophase I by the topoisomerase II-type enzyme Spo11 that becomes covalently linked to the 5′ end of each side of the DSB (Keeney *et al*, 1997). The two main DSB repair pathways are non-homologous end-joining (NHEJ) and homologous recombination (Lisby *et al*, 2004; Symington & Gautier, 2011). In NHEJ, DNA ends need little or no processing before being ligated (Daley *et al*, 2005). By contrast, HR requires DNA-end resection, a process involving degradation of the 5′ ends of the break, yielding 3′ single-stranded DNA (ssDNA) tails that mediate HR via pairing with and invading the sister chromatid, which provides the repair template.

Reflecting the above requirements, cells defective in resection components display HR defects and hypersensitivity to various DNA-damaging agents. This is well illustrated by *Saccharomyces cerevisiae* cells harbouring defects in the Mre11–Rad50–Xrs2 (MRX) complex, which binds and juxtaposes the two ends of a DSB (Williams *et al*, 2008) and, through Mre11 catalytic functions, provides nuclease activities involved in DSB processing (Furuse *et al*, 1998; Williams *et al*, 2008; Stracker & Petrini, 2011). Once a clean, partially resected 5′ end has been generated, the enzymes Exo1 and Sgs1/Dna2 are then thought to act, generating extensive ssDNA regions needed for effective HR (Mimitou & Symington, 2008; Zhu *et al*, 2008). Notably, while Mre11 nuclease activity is essential in meiosis to remove Spo11 and promote 5′ end resection, in mitotic cells, resection is only somewhat delayed in the absence of Mre11 and almost unaffected by *mre11-nd* (nuclease-dead) mutations (Ivanov *et al*, 1994; Moreau *et al*, 1999), indicating the existence of MRX-nuclease-independent routes for ssDNA generation.

Another protein linked to resection is *S. cerevisiae* Sae2, the functional homolog of human CtIP (Sartori *et al*, 2007; You *et al*, 2009). Despite lacking obvious catalytic domains, Sae2 and CtIP have been reported to display endonuclease activity *in vitro* (Lengsfeld *et al*, 2007; Makharashvili *et al*, 2014; Wang *et al*, 2014), and their functions are tightly regulated by cell cycle- and DNA damage-dependent phosphorylations (Baroni *et al*, 2004; Huertas *et al*, 2008; Huertas & Jackson, 2009; Barton *et al*, 2014). In many ways, Sae2 appears to function together with MRX in DSB repair. For instance, *mre11-nd* as well as *mre11S* and *rad50S* hypomorphic alleles phenocopy *SAE2* deletion (*sae2*Δ) in meiosis, yielding unprocessed Spo11–DNA complexes (Keeney & Kleckner, 1995; Nairz & Klein, 1997; Prinz *et al*, 1997). Furthermore, recent findings have indicated that Sae2 stimulates Mre11 endonuclease activity to promote resection, particularly at protein-bound DSB ends (Cannavo & Cejka, 2014). Also, both *sae2*Δ and *mre11-nd* mutations cause hypersensitivity towards the anti-cancer drug camptothecin (Deng *et al*, 2005), which yields DSBs that are repaired by HR. Nevertheless, key differences between MRX and Sae2 exist, since *sae2*Δ leads to persistence of MRX at DNA damage sites (Lisby *et al*, 2004) and hyperactivation of the MRX-associated Tel1 protein kinase (Usui *et al*, 2001), the homolog of human ATM, while MRX inactivation abrogates Tel1 function (Fukunaga *et al*, 2011). These findings, together with *sae2*Δ and *mre11-nd* cells displaying only mild resection defects (Clerici *et al*, 2005), highlight how Sae2 functions in HR cannot be readily explained by it simply cooperating with MRX to enhance resection.

As reported below, by combining classic genetic screening for suppressor mutants with whole-genome sequencing to determine their genotype, we are led to a model that resolves apparent paradoxes regarding Sae2 and MRX functions, namely the fact that while deletion of either *SAE2* or *MRE11* causes hypersensitivity to DNA-damaging agents, the resection defect of *sae2*Δ strains is negligible compared to that of *mre11*Δ cells, and lack of Sae2 causes an increase in Mre11 persistence at DSB ends rather than a loss. Our model invokes Mre11/MRX removal from DNA as a critical step in allowing HR to proceed effectively on a resected DNA template.

## Results

### SVGS identifies Mre11 mutations as *sae2*Δ suppressors

**Figure 1:**
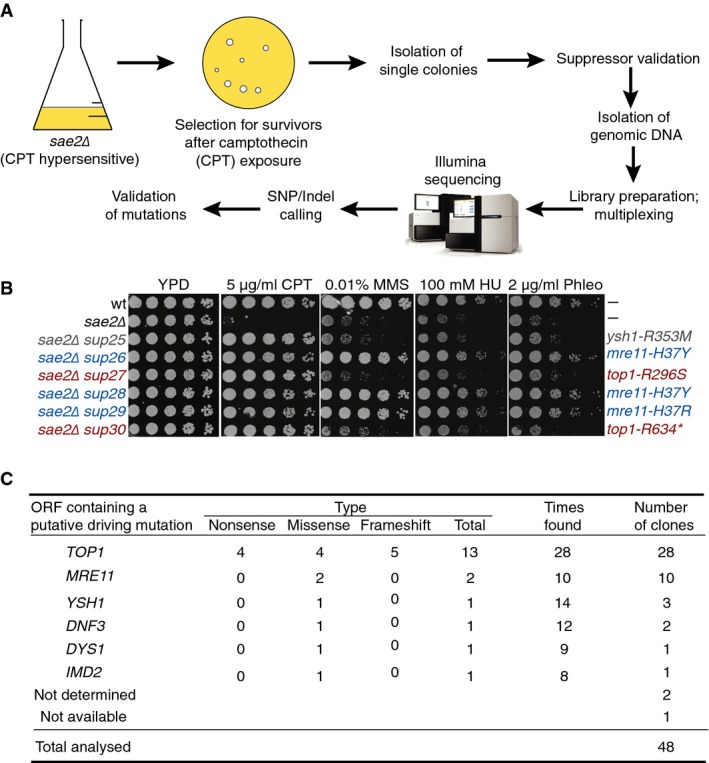
SVGS identifies mutations suppressing *sae2*Δ DNA damage hypersensitivity

To gain insights into why yeast cells lacking Sae2 are hypersensitive to DNA-damaging agents, we performed synthetic viability genomic screening (SVGS; Fig1A). To do this, we took cultures of a *sae2*Δ yeast strain (bearing a full deletion of the *SAE2* locus) and plated them on YPD plates supplemented with camptothecin, which stabilises DNA topoisomerase I cleavage complexes and yields replication-dependent DSBs that are repaired by Sae2-dependent HR (Deng *et al*, 2005) (Fig1A). Thus, we isolated 48 mutants surviving camptothecin treatment that spontaneously arose in the population analysed. In addition to verifying that all indeed contained the *SAE2* gene deletion yet were camptothecin resistant, subsequent analyses revealed that 10 clones were also largely or fully suppressed for *sae2*Δ hypersensitivity to the DNA-alkylating agent methyl methanesulphonate (MMS), the replication inhibitor hydroxyurea (HU), the DSB-generating agent phleomycin and ultraviolet light (Supplementary Fig S1).

Outline of the screening approach that was used to identify suppressors of *sae2*Δ camptothecin (CPT) hypersensitivity.

Validation of the suppression phenotypes; a subset (sup25–sup30) of the suppressors recovered from the screening is shown along with mutations identified in each clone.

Summary of the results of the synthetic viability genomic screening (SVGS) for *sae2*Δ camptothecin (CPT) hypersensitivity. The ORF and the type of mutation are reported together with the number of times each ORF was found mutated and the number of clones in which each ORF was putatively driving the resistance.

To identify mutations causing these suppression phenotypes, genomic DNA from the 48 clones was isolated and analysed by next-generation Illumina sequencing. We then used bioinformatics tools (see Materials and Methods) to identify mutations altering open reading frames within the reference *S. cerevisiae* genome (Fig1A). This revealed that 24 clones displaying camptothecin resistance but retaining *sae2*Δ hypersensitivity towards other DNA-damaging agents possessed *TOP1* mutations (Fig1B and C), thereby providing proof-of-principle for the SVGS methodology (*TOP1* is a non-essential gene that encodes DNA topoisomerase I, the camptothecin target). Strikingly, of the remaining clones, 10 contained one or other of two different mutations in a single *MRE11* codon, resulting in amino acid residue His37 being replaced by either Arg or Tyr (*mre11-H37R* and *mre11-H37Y,* respectively; Fig1B and C and Supplementary Fig S1; note that *TOP1* and *MRE11* mutations are mutually exclusive). While some remaining clones contained additional potential suppressor mutations worthy of further examination, these were only resistant to camptothecin. Because of their broader phenotypes and undefined mechanism of action, we focused on characterising the *MRE11 sae2*Δ suppressor (*mre11*^*SUPsae2*Δ^) alleles.

### *mre11*^*SUPsae2*Δ^ alleles suppress many *sae2*Δ phenotypes

**Figure 2:**
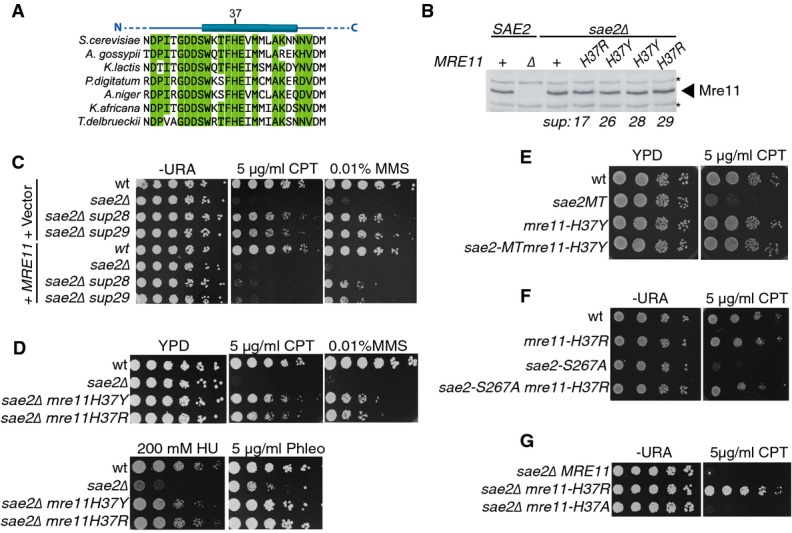
*mre11-H37R* suppresses the CPT hypersensitivity of *sae2*Δ cells

Mre11 His37 lies within a functionally undefined but structurally evolutionarily conserved α-helical region, and the residue is well conserved among quite divergent fungal species (Fig2A). As anticipated from previous studies, deleting *MRE11* did not suppress the DNA damage hypersensitivities of *sae2*Δ cells, revealing that *mre11-H37R* and *mre11-H37Y* were not behaving as null mutations (unpublished observation). In line with this, the *mre11-H37R* and *mre11-H37Y* alleles did not destabilise Mre11, producing proteins that were expressed at equivalent levels to the wild-type protein (Fig2B). Nevertheless, expression of wild-type Mre11 resensitised the *mre11*^*SUPsae2*Δ^ *sae2*Δ strains to camptothecin, and to a lesser extent to MMS (Fig2C), indicating that *mre11-H37R* and *mre11-H37Y* were fully or partially recessive for the camptothecin and MMS resistance phenotypes, respectively. Furthermore, this established that expression of wild-type Mre11 is toxic to *sae2*Δ*mre11*^*SUPsae2*Δ^ cells upon camptothecin treatment. Importantly, independent introduction of *mre11-H37R* and *mre11-H37Y* alleles in a *sae2*Δ strain confirmed that each conferred suppression of *sae2*Δ hypersensitivity to various DNA-damaging agents (Fig2D). The *mre11-H37R* and *mre11-H37Y* alleles also suppressed camptothecin hypersensitivity caused by mutations in Sae2 that prevent its Mec1/Tel1-dependent (*sae2-MT*) or CDK-dependent (*sae2-S267A)* phosphorylation (Baroni *et al*, 2004; Huertas *et al*, 2008) (Fig2E and F). By contrast, no suppression of *sae2*Δ camptothecin hypersensitivity was observed by mutating His37 to Ala (*mre11-H37A*; Fig2G), suggesting that the effects of the *mre11*^*SUPsae2*Δ^ alleles were not mediated by the abrogation of a specific function of His37 but more likely reflected functional alteration through introducing bulky amino acid side chains.

A Alignment of Mre11 region containing H37 in fungal species; secondary structure prediction is shown above.

B Western blot with anti-Mre11 antibody on protein extracts prepared from the indicated strains shows that *mre11-H37R* and *mre11*-*H37Y* mutations do not alter Mre11 protein levels (* indicate cross-reacting proteins).

C *sup28* and *sup29* suppression is rescued by expressing wild-type (wt) Mre11.

D *mre11-H37R* and *mre11-H37Y* suppress *sae2*Δ DNA damage hypersensitivity.

E, F *mre11-H37Y* suppresses DNA damage hypersensitivities of *sae2MT (sae2-2,5,6,8,9)* and *sae2-S267A* cells. CPT, camptothecin; Phleo, phleomycin.

G *mre11-H37A* does not suppress *sae2*Δ.

### *mre11*^*SUPsae2*Δ^ alleles do not suppress all *sae2*Δ phenotypes

**Figure 3:**
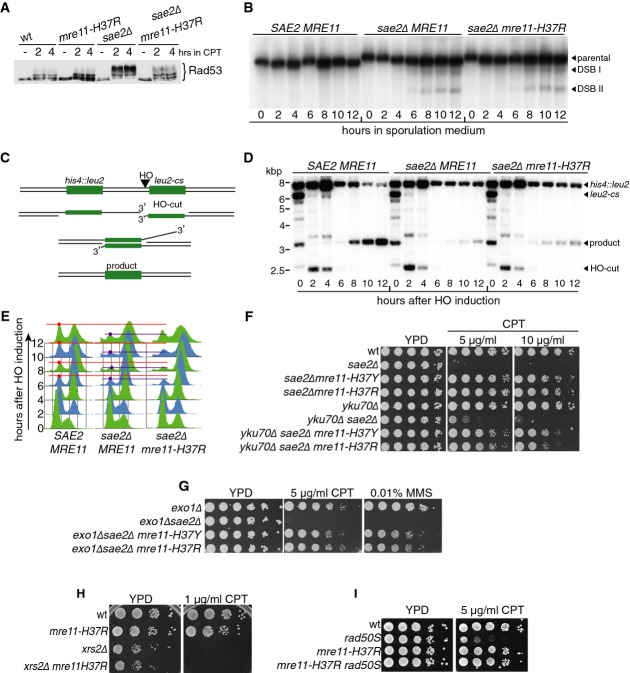
*mre11-H37R* suppresses some but not all *sae2*Δ phenotypes

In the absence of Sae2, cells display heightened DNA damage signalling as measured by Rad53 hyperphosphorylation (Clerici *et al*, 2006). As we had found for the DNA damage hypersensitivities of *sae2*Δ cells, this read-out of Sae2 inactivity was also rescued by *mre11-H37R* (Fig3A). By contrast, *mre11-H37R* did not suppress the sporulation defect of *sae2*Δ cells (unpublished observation). In line with this, *mre11-H37R* did not suppress impaired meiotic DSB processing caused by Sae2 deficiency, as reflected by aberrant accumulation of 5′-bound Spo11 repair intermediates within the *THR4* recombination hot spot (Goldway *et al*, 1993; Fig3B; as shown in Supplementary Fig S2A, *mre11-H37R* did not itself cause meiotic defects when Sae2 was present). Notably, however, *mre11-H37R* rescued the hypersensitivity of *sae2*Δ cells to etoposide, which produces DSBs bearing 5′ DNA ends bound to Top2 (Supplementary Fig S2B; deletion of *ERG6* was used to increase permeability of the plasma membrane to etoposide), suggesting that significant differences must exist between the repair of meiotic and etoposide-induced DSBs.

A *mre11-H37R* suppresses *sae2*Δ checkpoint hyperactivation.

B *mre11-H37R* does not rescue *sae2*Δ meiotic DSB processing defect.

C Outline of DSB repair by single-strand annealing (SSA).

D *mre11-H37R* does not rescue the SSA repair defect of *sae2*Δ strains.

E *mre11-H37R* does not rescue *sae2*Δ-dependent cell cycle arrest caused by DSB induction.

F, G Exo1 and Ku are not required for *mre11-H37R*-mediated suppression of *sae2*Δ hypersensitivity.

H *mre11-H37R* does not suppress *xrs2*Δ camptothecin (CPT) hypersensitivity.

I *mre11-H37R* suppresses *rad50S* CPT hypersensitivity.

Next, we examined the effects of *mre11*^*SUPsae2*Δ^ alleles on Sae2-dependent DSB repair by single-strand annealing (SSA), using a system wherein a chromosomal locus contains an HO endonuclease cleavage site flanked by two direct sequence repeats. In this system, HO induction produces a DSB that is then resected until two complementary sequences become exposed and anneal, resulting in repair by a process that deletes the region between the repeats (Fishman-Lobell *et al*, 1992; Vaze *et al*, 2002; Fig3C). Despite displaying only mild resection defects (Clerici *et al*, 2006), we observed that *sae2*Δ cells were defective in SSA-mediated DSB repair and did not resume cell cycle progression after HO induction as fast as wild-type cells, in agreement with published work (Clerici *et al*, 2005). Notably, *mre11-H37R* did not alleviate these *sae2*Δ phenotypes (Fig3D and E).

Finally, we examined the effect of the *mre11-H37R* mutation on telomere-associated functions of the MRX complex and Sae2. It has been established that simultaneous deletion of *SGS1* and *SAE2* results in synthetic lethality/sickness, possibly due to excessive telomere shortening (Mimitou & Symington, 2008; Hardy *et al*, 2014). To test whether *mre11-H37R* can alleviate this phenotype, we crossed a *sae2*Δ*mre11-H37R* strain with a *sgs1*Δ strain. As shown in Supplementary Fig S2C, we were unable to recover neither *sgs1*Δ*sae2*Δ nor *sgs1*Δ*sae2*Δ*mre11-H37R* cells, implying that *mre11-H37R* cannot suppress this phenotype. In agreement with this conclusion, the *mre11-H37R* mutation did not negatively affect Mre11-dependent telomere maintenance as demonstrated by Southern blot analysis (Supplementary Fig S2D).

Together, the above data revealed that *mre11*^*SUPsae2*Δ^ alleles suppressed *sae2*Δ DNA damage hypersensitivities but not *sae2*Δ meiotic phenotypes requiring Mre11-mediated Spo11 removal from recombination intermediates, nor mitotic SSA functions that have been attributed to Sae2-mediated DNA-end bridging (Clerici *et al*, 2005). Subsequent analyses revealed that suppression did not arise largely through channelling of DSBs towards NHEJ because the key NHEJ factor Yku70 was not required for *mre11-H37R* or *mre11-H37Y* to suppress the camptothecin sensitivity of a *sae2*Δ strain (Fig3F). In addition, this analysis revealed that the previously reported suppression of *sae2*Δ-mediated DNA damage hypersensitivity by Ku loss (Mimitou & Symington, 2010; Foster *et al*, 2011) was considerably less effective than that caused by *mre11-H37R* or *mre11-H37Y*. Also, suppression of *sae2*Δ camptothecin hypersensitivity by *mre11*^*SUPsae2*Δ^ alleles did not require Exo1, indicating that in contrast to suppression of *sae2*Δ phenotypes by Ku loss (Mimitou & Symington, 2010), *mre11-H37R* and *mre11-H37Y* did not cause cells to become particularly reliant on Exo1 for DSB processing (Fig3G). Further characterisations, focused on *mre11-H37R*, revealed that while not suppressing camptothecin hypersensitivity of an *xrs2*Δ strain (Fig3H), it almost fully rescued the camptothecin hypersensitivity of a strain expressing the *rad50S* allele, which phenocopies *sae2*Δ by somehow preventing functional Sae2–MRX interactions that are required for Sae2 stimulation of Mre11 endonuclease activity (Keeney & Kleckner, 1995; Hopfner *et al*, 2000; Cannavo & Cejka, 2014; Fig3I).

### H37R does not enhance Mre11 nuclease activity but impairs DNA binding

**Figure 4:**
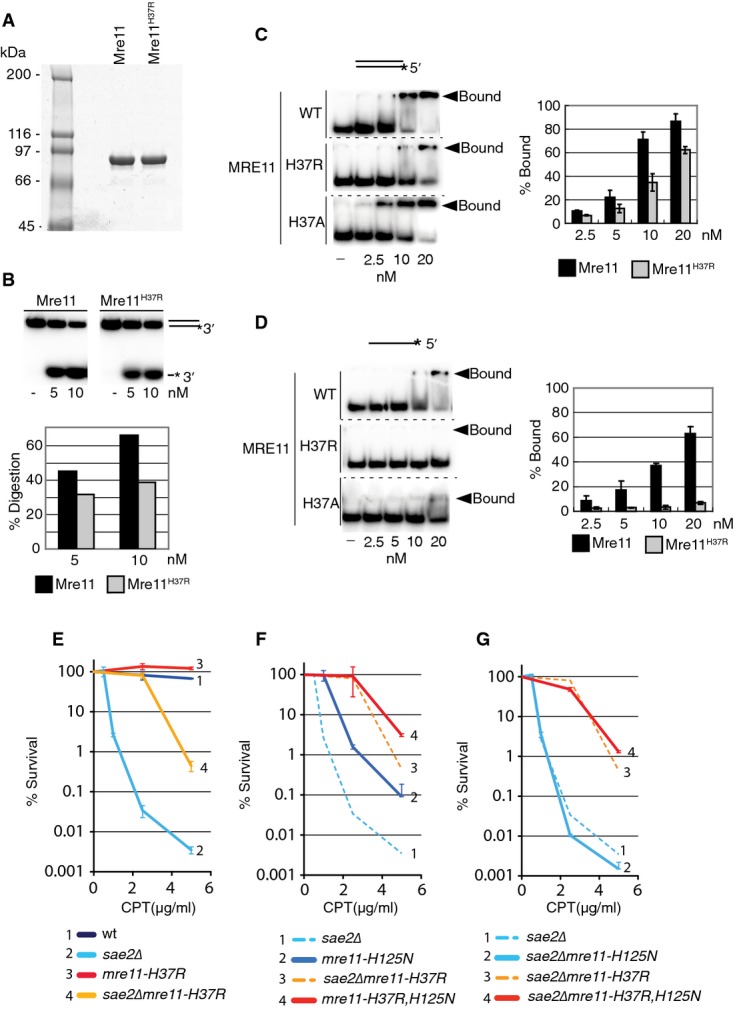
Mre11^H37R^ is impaired biochemically, particularly at the level of ssDNA binding

To explore how *mre11*^*SUPsae2*Δ^ mutations might operate, we over-expressed and purified wild-type Mre11, Mre11^H37R^ and Mre11^H37A^ (Fig4A and Supplementary Fig S2F) and then subjected these to biochemical analyses. All the proteins were expressed at similar levels and fractionated with equivalent profiles, suggesting that the Mre11 mutations did not grossly affect protein structure or stability. Since Sae2 promotes Mre11 nuclease functions, we initially speculated that *sae2*Δ suppression would be mediated by *mre11*^*SUPsae2*Δ^ alleles having intrinsically high, Sae2-independent nuclease activity. Surprisingly, this was not the case, with Mre11^H37R^ actually exhibiting lower nuclease activity than the wild-type protein (Fig4B). Furthermore, by electrophoretic mobility shift assays, we found that the H37R mutation reduced Mre11 binding to double-stranded DNA (dsDNA; Fig4C) and abrogated Mre11 binding to ssDNA (Fig4D). Conversely, mutation of H37 to alanine, which does not result in a *sup*^*sae2*Δ^ phenotype*,* did not negatively affect dsDNA-binding activity (Fig4C) and only partially impaired ssDNA binding (Fig4D).

A Mre11 and Mre11^H37R^ were purified to homogeneity from yeast cultures.

B 3′ exonuclease activity assay on Mre11 and Mre11^H37R^ leading to release of a labelled single nucleotide, as indicated.

C, D Electrophoretic mobility shift assays on Mre11, Mre11^H37R^ and Mre11^H37A^ with dsDNA (C) or ssDNA (D).

E Quantification of mre11-H37R suppression of *sae2*Δ cell DNA damage hypersensitivity. Overnight grown cultures of the indicated strains were diluted and plated on medium containing the indicated doses of CPT. Colony growth was scored 3–6 days later. Averages and standard deviations are shown for each point.

F Intragenic suppression of CPT hypersensitivity of *mre11-nd* (*mre11-H125N*) by *mre11-H37R*. Overnight grown cultures of the indicated strains were treated as in (E). Dotted lines represent data from (E). Averages and standard deviations are shown for each point.

G Mre11 nuclease activity is not required for *mre11-H37R*-mediated suppression of *sae2*Δ CPT hypersensitivity. Overnight grown cultures of the indicated strains were treated as in (E). The dotted lines represent data from (E). Averages and standard deviations are shown for each point.

Taken together with the fact that the lack of Sae2 only has minor effects on mitotic DSB resection (Clerici *et al*, 2005), the above results suggested that the *sae2*Δ suppressive effects of *mre11*^*SUPsae2*Δ^ mutations were associated with weakened Mre11 DNA binding and were not linked to effects on resection or Mre11 nuclease activity. In line with this idea, by combining mutations in the same Mre11 polypeptide, we established that *mre11-H37R* substantially rescued camptothecin hypersensitivity caused by mutating the Mre11 active site residue His125 to Asn (Moreau *et al*, 2001; *mre11-H125N*; Fig4E and Supplementary Fig S2F and G), which abrogates all Mre11 nuclease activities and prevents processing of DSBs when their 5′ ends are blocked (Moreau *et al*, 1999). Even *sae2*Δ *mre11-H37R,H125N* cells were resistant to camptothecin and MMS, indicating that Mre11-nuclease-mediated processing of DNA ends is not required for H37R-dependent suppression, nor for DNA repair in this Sae2-deficient setting (Fig4G and Supplementary Fig S2G). Furthermore, while *sae2*Δ strains were more sensitive to camptothecin than *mre11-H125N* strains, the sensitivities of the corresponding strains carrying the *mre11-H37R* allele were comparable (compare curves 1 and 2 with 3 and 4 in Fig4F) indicating that *mre11-H37R* suppresses not only the *sae2*Δ-induced lack of Mre11 nuclease activity, but also other nuclease-independent functions of Sae2. Nevertheless, *mre11-H37R* did not rescue the camptothecin hypersensitivity of *sae2*Δ cells to wild-type levels, suggesting that not all functions of Sae2 are suppressed by this *MRE11* allele (Fig4E and F).

### Identifying an Mre11 interface mediating *sae2*Δ suppression

**Figure 5:**
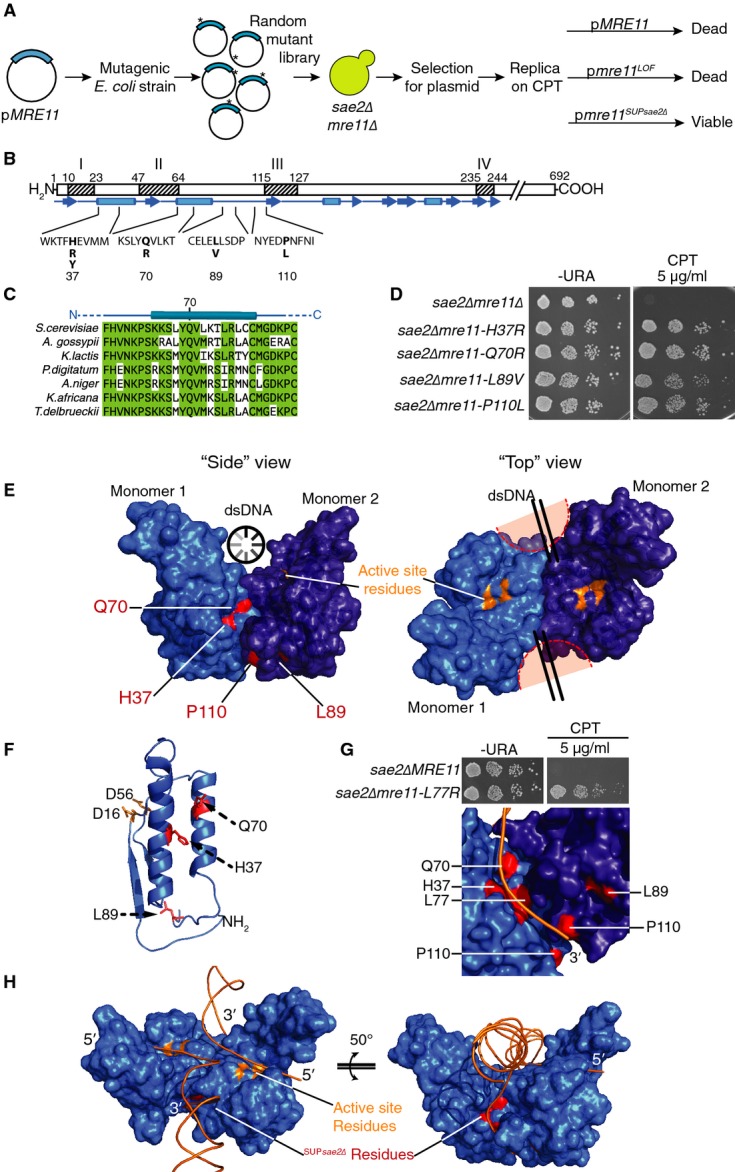
Identifying additional mutations in *MRE11* that mediate *sae2*Δ suppression

To gain further insights into how *mre11*^*SUPsae2*Δ^ alleles operate and relate this to the above functional and biochemical data, we screened for additional *MRE11* mutations that could suppress camptothecin hypersensitivity caused by Sae2 loss. Thus, we propagated a plasmid carrying wild-type *MRE11* in a mutagenic *E. coli* strain, thereby generating libraries of plasmids carrying *mre11* mutations. We then introduced these libraries into a *sae2*Δ*mre11*Δ strain and screened for transformants capable of growth in the presence of camptothecin (Fig5A). Through plasmid retrieval, sequencing and functional verification, we identified 12 *sae2*Δ suppressors, nine carrying single *mre11* point mutations and three being double mutants (Supplementary Fig S3A). One single mutant was *mre11-H37R*, equivalent to an initial spontaneously arising suppressor that we had identified. Among the other single mutations were *mre11-P110L* and *mre11-L89V*, both of which are located between Mre11 nuclease domains II and III, in a region with no strong secondary structure predictions (Fig5B). Two of the three double mutants contained *mre11-P110L* combined with another mutation that was presumably not responsible for the resistance phenotype (because *mre11-P110L* acts as a suppressor on its own), whereas the third contained both *mre11-Q70R* and *mre11-G193S*. Subsequent studies, involving site-directed mutagenesis, demonstrated that effective *sae2*Δ suppression was mediated by *mre11-Q70R*, which alters a residue located in a highly conserved α-helical region (Fig5C). Ensuing comparisons revealed that the mutations identified did not alter Mre11 protein levels (Supplementary Fig S3B) and that *mre11-Q70R* suppressed *sae2*Δ camptothecin hypersensitivity to similar extents as *mre11-H37R* and *mre11-H37Y*, whereas *mre11-L89V* and *mre11-P110L* were marginally weaker suppressors (Fig5D).

Outline of the plasmid mutagenesis approach to identify new *mre11*^*SUPsae2*Δ^ alleles. ^*LOF*^: loss-of-function alleles. ^*SUP*^: suppressor alleles.

Mre11 with shaded boxes and blue shapes indicating phosphoesterase motifs and secondary structures, respectively; additional *mre11*^*SUPsae2*Δ^ mutations recovered from the screening are indicated.

Fungal alignment and secondary structure prediction of the region of Mre11 containing Q70.

*mre11-Q70R, mre11-L89V* and *mre11-P110L* alleles recovered from plasmid mutagenesis screening suppress *sae2*Δ hypersensitivity to camptothecin.

Structural prediction of *S. cerevisiae* Mre11 residues 1–414, obtained by homology modelling using the corresponding *S. pombe* and human structures. The water-accessible surface of the two monomers is shown in different shades of blue. Red: residues whose mutation suppresses *sae2*Δ DNA damage hypersensitivity. Orange: residues whose mutation abrogates Mre11 nuclease activity.

Model of Mre11 tertiary structure (residues 1–100). Residues are colour-coded as in (E).

Top: *mre11-L77R* suppresses the DNA damage hypersensitivity of *sae2*Δ cells. Bottom: localisation of *mre11*^*SUPsae2*Δ^ suppressors on the molecular model of the Mre11 dimer. The two Mre11 monomers are shown in different shades of blue, and the proposed path of bound ssDNA is indicated by the orange filament.

Model in which the two DNA filaments of the two DSB ends melt when binding to Mre11; the 5′ ends being channelled towards the active site and the 3′ end being channelled towards the Mre11^SUPsae2Δ^ region.

To map the locations of the various *mre11*^*SUPsae2*Δ^ mutations within the Mre11 structure, we used the dimeric tertiary structure (Schiller *et al*, 2012) of the *Schizosaccharomyces pombe* Mre11 counterpart, Rad32, as a template to generate a molecular model of *S. cerevisiae* Mre11. The resulting structure had a near-native QMEAN score (0.705 vs 0.778; Benkert *et al*, 2008), indicating a reliable molecular model. Strikingly, ensuing analyses indicated that the *mre11*^*SUPsae2*Δ^ mutations clustered in a region of the protein structure distal from the nuclease catalytic site and adjacent to, but distinct from, the interface defined as mediating contacts with dsDNA in the *Pyrococcus furiosus* Mre11 crystal structure (Williams *et al*, 2008; Fig5E; the predicted path of dsDNA is shown in black, while the *mre11*^*SUPsae2*Δ^ mutations and residues involved in nuclease catalysis are indicated in red and orange, respectively). Furthermore, this analysis indicated that H37 and Q70 are located close together, on two parallel α-helices and are both likely to be solvent exposed (Fig5F). By contrast, the L89 side chain is predicted to be in the Mre11 hydrophobic core, although modelling suggested that the *mre11-L89V* mutation might alter the stability of the α-helix containing Q70. We noted that, in the context of the Mre11 dimer, H37 and Q70 are located in a hemi-cylindrical concave area directly below the position where dsDNA is likely to bind (Fig5E right, shown by pink hemispheres). Furthermore, by specifically mutating other nearby residues to arginine, we found that the *mre11-L77R* mutation also strongly suppressed *sae2*Δ camptothecin hypersensitivity (Fig5G). As discussed further below, while it is possible that certain *mre11*^*SUPsae2*Δ^ alleles somehow influence the established dsDNA-binding interface of Mre11, we speculate that *mre11-H37R/Y* and *mre11-Q70R,* and at least some of the other suppressors, act by perturbing interactions normally mediated between the Mre11 hemi-cylindrical concave region and ssDNA (modelled in Fig5G and discussed further below). Consistent with this idea, we found that the Mre11^Q70R^ protein was markedly impaired in binding to ssDNA but not to dsDNA (Supplementary Figs S2E and S3C). However, because P110 lies in the ‘latching loop’ region of eukaryotic Mre11 that is likely to mediate contacts with Xrs2 (Schiller *et al*, 2012), *sae2*Δ suppression by this mutation might arise through altering such contacts. A recent report by L. Symington and colleagues reached similar conclusions (Chen *et al*, 2015).

Taken together, our findings suggested that, in addition to its established dsDNA-binding mode, Mre11 mediates distinct, additional functional contacts with DNA that, when disrupted, lead to suppression of *sae2*Δ phenotypes. Thus, we suggest that, during DSB processing, duplex DNA entering the Mre11 structure may become partially unwound, with the 5′ end being channelled towards the nuclease catalytic site and the resulting ssDNA—bearing the 3′ terminal OH—interacting with an adjacent Mre11 region that contains residues mutated in *mre11*^*SUPsae2*Δ^ alleles (Fig5G and H). In this regard, we note that Mre11 was recently shown in biochemical studies to promote local DNA unwinding (Cannon *et al*, 2013). Such a model would explain our biochemical findings, and would also explain our biological data if persistent Mre11 binding to the nascent 3′ terminal DNA impairs HR unless counteracted by the actions of Sae2 or weakened by *mre11*^*SUPsae2*Δ^ alleles.

### *sae2*Δ phenotypes reflect Mre11-bound DNA repair intermediates

**Figure 6:**
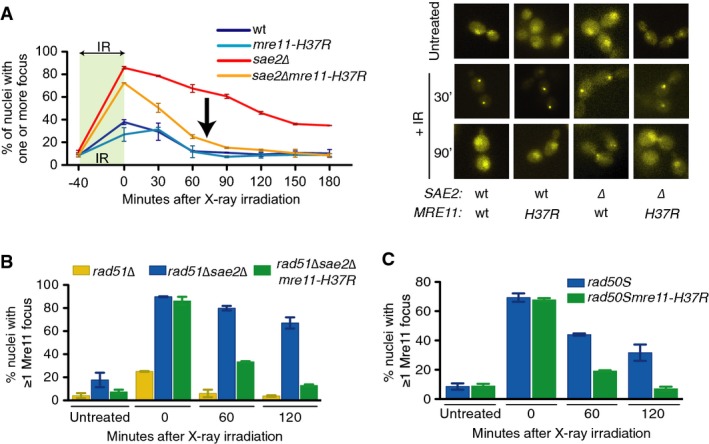
*mre11*^*SUPsae2*Δ^ alleles bypass the need for Sae2 to remove Mre11 from DSB ends

A prediction arising from the above model is that Mre11 persistence and associated Tel1 hyperactivation in *sae2*Δ cells would be counteracted by *mre11*^*SUPsae2*Δ^ mutations. To test this, we constructed yeast strains expressing wild-type Mre11 or Mre11^H37R^ fused to yellow-fluorescent protein (YFP) and then used fluorescence microscopy to examine their recruitment and retention at sites of DNA damage induced by ionising radiation. In line with published work (Lisby *et al*, 2004), recruitment of wild-type Mre11 to DNA damage foci was more robust and persisted longer when Sae2 was absent (Fig6A). Moreover, such Mre11 DNA damage persistence in *sae2*Δ cells was largely attenuated by *mre11-H37R* (Fig6A; compare red and orange curves). By contrast, *mre11-H37R* had little or no effect on Mre11 recruitment and dissociation kinetics when Sae2 was present (compare dark and light blue curves). Importantly, we found that HR-mediated DSB repair was not required for H37R-induced suppression of Mre11-focus persistence in *sae2*Δ cells, as persistence and suppression still occurred in the absence of the key HR factor, Rad51 (Fig6B). Also, in accord with our other observations, we found that the *rad50S* allele caused Mre11 DNA damage-focus persistence in a manner that was suppressed by the *mre11-H37R* mutation (Fig6C).

IR-induced Mre11^H37R^ foci (IRIF) persist for shorter times than Mre11-wt IRIF in exponentially growing *sae2*Δ cells (average and standard deviations from two or more independent experiments).

Effects of *sae2*Δ and *mre11-H37R* on Mre11 IRIF persistence still occur when Rad51 is absent, revealing that Mre11 IRIF persistence causes defective HR (average and standard deviation from two independent experiments).

*mre11-H37R* suppresses Mre11 IRIF persistence in exponentially growing *rad50S* cells (average and standard deviation from two independent experiments).

**Figure 7:**
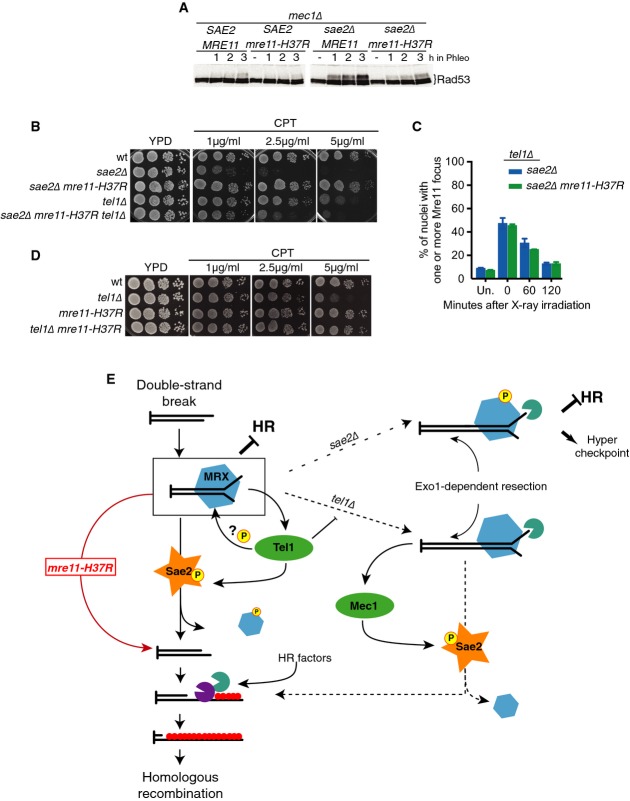
Tel1 participates in regulating Mre11 dynamics after DNA damage

Previous work has established that Mre11 persistence on DSB ends, induced by lack of Sae2, leads to enhanced and prolonged DNA damage-induced Tel1 activation, associated with Rad53 hyperphosphorylation (Usui *et al*, 2001; Lisby *et al*, 2004; Clerici *et al*, 2006; Fukunaga *et al*, 2011). Supporting our data indicating that, unlike wild-type Mre11, Mre11^H37R^ is functionally released from DNA ends even in the absence of Sae2, we found that in a *mec1*Δ background (in which Tel1 is the only kinase activating Rad53; Sanchez *et al*, 1996), DNA damage-induced Rad53 hyperphosphorylation was suppressed by *mre11-H37R* (Fig7A).

*mre11-H37R* suppresses Tel1 hyperactivation induced by Mre11 IRIF persistence in *sae2*Δ cells.

Deletion of *TEL1* weakens the suppression of the sensitivity of a *sae2*Δ strain mediated by *mre11-H37R*.

Deletion of *TEL1* reduces the hyperaccumulation of Mre11 to IRIF and impairs the suppression of their persistence mediated by *mre11-H37R* (average and standard deviation from two independent experiments).

*mre11-H37R* suppresses the sensitivity to CPT of a *tel1*Δ strain.

Model for the role of MRX, Sae2 and Tel1 in response to DSBs.

While we initially considered the possibility that persistent Tel1 hyperactivation might cause the DNA damage hypersensitivity of *sae2*Δ cells, we concluded that this was unlikely to be the case because *TEL1* inactivation did not suppress *sae2*Δ DNA damage hypersensitivity phenotypes (Supplementary Fig S3D). Furthermore, Tel1 loss actually reduced the ability of *mre11-H37R* to suppress the camptothecin hypersensitivity of *sae2*Δ cells (Fig7B). In accord with this, in the absence of Tel1, *mre11-H37R* no longer affected the dissociation kinetics of IR-induced Mre11 foci in *sae2*Δ cells (Fig7C). Collectively, these data suggested that Tel1 functionally cooperates with Sae2 to promote the removal of Mre11 from DNA ends. In this regard, we noted that *mre11-H37R* suppressed the moderate camptothecin hypersensitivity of a *tel1*Δ strain (Fig7D). We therefore propose that, while persistent DNA damage-induced Tel1 activation is certainly a key feature of *sae2*Δ cells, it is persistent binding of the MRX complex to nascent 3′ terminal DNA that causes toxicity in *sae2*Δ cells, likely through it delaying downstream HR events. Accordingly, mutations that reduce Mre11 ssDNA binding enhance the release of the Mre11 complex from DSB ends in the absence of Sae2, through events promoted by Tel1 (Fig7E). In this model, Mre11 persistence at DNA damage sites is a cause, and not just a consequence, of impaired HR-mediated repair in sae2Δ cells.

## Discussion

Our data help resolve apparent paradoxes regarding Sae2 and MRX function by suggesting a revised model for how these and associated factors function in HR (Fig7E). In this model, after being recruited to DSB sites and promoting Tel1 activation, resection and ensuing Mec1 activation, the MRX complex disengages from processed DNA termini in a manner promoted by Sae2 and facilitated by Tel1 and Mre11 nuclease activity. Sae2 is required to stimulate Mre11 nuclease activity (Cannavo & Cejka, 2014) and subsequently to promote MRX eviction from the DSB end. However, our data suggest that Sae2 can also promote MRX eviction in the absence of DNA-end processing, as *mre11-H37R* suppresses the phenotypes caused by *sae2*Δ and *mre11-nd* to essentially the same extent. Thus, according to our model, when Sae2 is absent, both the nuclease activities of Mre11 and MRX eviction are impaired. Under these circumstances, despite resection taking place—albeit with somewhat slower kinetics than in wild-type cells—MRX persists on ssDNA bearing the 3′ terminal OH, thereby delaying repair by HR. In cells containing the *mre11-H37R* mutation, however, weakened DNA binding together with Tel1 activity promotes MRX dissociation from DNA even in the absence of Sae2, thus allowing the nascent ssDNA terminus to effectively engage in the key HR events of strand invasion and DNA synthesis (Fig7E). Nevertheless, it is conceivable that abrogation of pathological Tel1-mediated checkpoint hyperactivation contributes to the resistance of *sae2*Δ*mre11-H37R* cells to DNA-damaging agents. In this regard, we note that the site of one of the *sae2*Δ suppressors, P110, lies in the ‘latching loop’ region of eukaryotic Mre11 that is likely to mediate contacts with Xrs2 (Schiller *et al*, 2012), suggesting that, in this case, *sae2*Δ suppression might arise through weakening this interaction and dampening Tel1 activity.

Our results also highlight how the camptothecin hypersensitivity of strains carrying a nuclease-defective version of Mre11 does not reflect defective Mre11-dependent DNA-end processing *per se*, but rather stems from stalling of MRX on DNA ends. We propose that this event delays or prevents HR, possibly by impairing the removal of 3′-bound Top1 as is suggested by the fact that in *S. pombe, rad50S* or *mre11-nd* alleles are partially defective in Top1 removal from damaged DNA (Hartsuiker *et al*, 2009). This interpretation also offers an explanation for the higher DNA damage hypersensitivity of *sae2*Δ cells compared to cells carrying *mre11-H125N* alleles: while *sae2*Δ cells are impaired in both Mre11 nuclease activity and Mre11 eviction—leading to MRX persistence at DNA damage sites and Tel1 hyperactivation—*mre11-H125N* cells are only impaired in Mre11 nuclease activity. Indeed, despite having no nuclease activity, the *mre11-H125N* mutation does not impair NHEJ, telomere maintenance, mating type switching or Mre11 interaction with Rad50/Xrs2 or interfere with the recruitment of the Mre11–Rad50–Xrs2 complex to foci at sites of DNA damage (Moreau *et al*, 1999; Lisby *et al*, 2004; Krogh *et al*, 2005). In addition, our model explains why the *mre11-H37R* mutation does not suppress meiotic defects of *sae2*Δ cells, because Sae2-stimulated Mre11 nuclease activity is crucial for removing Spo11 from meiotic DBS 5′ termini. Finally, this model explains why *mre11-H37R* does not suppress the *sae2*Δ deficiency in DSB repair by SSA because the *sae2*Δ defect in SSA is suggested to stem from impaired bridging between the two ends of a DSB rather than from the persistence of MRX on DNA ends (Clerici *et al*, 2005; Andres *et al*, 2015; Davies *et al*, 2015). In this regard, we note that SSA does not require an extendable 3′-OH DNA terminus to proceed and so could ensue even in the presence of blocked 3′-OH DNA ends.

We have also found that the *mre11-H37R* mutation suppresses the DNA damage hypersensitivities of cells impaired in CDK- or Mec1/Tel1-mediated Sae2 phosphorylation. This suggests that such kinase-dependent control mechanisms—which may have evolved to ensure that HR only occurs after the DNA damage checkpoint has been triggered—also operate, at least in part, at the level of promoting MRX removal from partly processed DSBs. Accordingly, we found that *TEL1* deletion causes moderate hypersensitivity to camptothecin that can be rescued by the *mre11-H37R* allele, implying that the same type of toxic repair intermediate is formed in *sae2*Δ and *tel1*Δ cells and that in each case, this can be rescued by MRX dissociation caused by *mre11-H37R* (Fig7E). Supporting this idea, it has been previously shown that resection relies mainly on Exo1 in both *tel1*Δ and *sae2*Δ cells (Clerici *et al*, 2006; Mantiero *et al*, 2007). We suggest that the comparatively mild hypersensitivity of *tel1*Δ strains to camptothecin is due to Tel1 loss allowing DSB repair intermediates to be channelled into a different pathw ay, in which Exo1-dependent resection (Mantiero *et al*, 2007) leads to the activation of Mec1, which can then promote Sae2 phosphorylation and subsequent MRX removal (Fig7E). The precise role of Tel1 in these events is not yet clear, although during the course of our analyses, we found that the deletion of *TEL1* reduced the suppressive effects of *mre11-H37R* on *sae2*Δ DNA damage sensitivity and Mre11-focus persistence. This suggests that, in the absence of Sae2, Tel1 facilitates MRX eviction by *mre11-H37R*, possibly by phosphorylating the MRX complex itself.

Given the apparent strong evolutionary conservation of Sae2, the Mre11–Rad50–Xrs2 complex and their associated control mechanisms, it seems likely that the model we have proposed will also apply to other systems, including human cells. Indeed, we speculate the profound impacts of proteins such as mammalian CtIP and BRCA1 on HR may not only relate to their effects on resection but may also reflect them promoting access to ssDNA bearing 3′ termini so that HR can take place effectively. Finally, our data highlight the power of SVGS to identify genetic interactions—including those such that we have defined that rely on separation-of-function mutations rather than null ones—and also to inform on underlying biological and biochemical mechanisms. In addition to being of academic interest, such mechanisms are likely to operate in medical contexts, such as the evolution of therapy resistance in cancer.

## Materials and Methods

### Strain and plasmid construction

Yeast strains used in this work are derivatives of SK1 (meiotic phenotypes), YMV80 (SSA phenotypes) and haploid derivatives of W303 (all other phenotypes). All deletions were introduced by one-step gene disruption. pRS303-derived plasmids, carrying a wt or mutant *MRE11* version, were integrated at the *MRE11* locus in an *mre11*Δ*::KanMX6* strain. Alternatively, the same strain was transformed with pRS416-derived plasmids containing wild-type or mutant *MRE11* under the control of its natural promoter. Strains expressing mutated *mre11-YFP* were obtained in two steps: integration of a pRS306-based plasmid (pFP118.1) carrying a mutated version of Mre11 in a *MRE11-YPF sae2*Δ strain, followed by selection of those ‘pop-out’ events that suppressed camptothecin hypersensitivity of the starting strain. The presence of mutations was confirmed by sequencing. Full genotypes of the strains used in this study are described in Supplementary Table S1; plasmids are described in Supplementary Table S2.

### Whole-genome paired-end DNA sequencing and data analysis

DNA (1–3 μg) was sheared to 100–1,000 bp by using a Covaris E210 or LE220 (Covaris, Woburn, MA, USA) and size-selected (350–450 bp) with magnetic beads (Ampure XP; Beckman Coulter). Sheared DNA was subjected to Illumina paired-end DNA library preparation and PCR-amplified for six cycles. Amplified libraries were sequenced with the HiSeq platform (Illumina) as paired-end 100 base reads according to the manufacturer's protocol. A single sequencing library was created for each sample, and the sequencing coverage per sample is given in Supplementary Table S3. Sequencing reads from each lane were aligned to the *S. cerevisiae* S288c assembly (R64-1-1) from *Saccharomyces* Genome Database (obtained from the Ensembl genome browser) by using BWA (v0.5.9-r16) with the parameter ‘-q 15’. All lanes from the same library were then merged into a single BAM file with Picard tools, and PCR duplicates were marked by using Picard ‘MarkDuplicates’ (Li *et al*, 2009). All of the raw sequencing data are available from the ENA under accession ERP001366. SNPs and indels were identified by using the SAMtools (v0.1.19) mpileup function, which finds putative variants and indels from alignments and assigns likelihoods, and BCFtools that performs the variant calling (Li *et al*, 2009). The following parameters were used: for SAMtools (v0.1.19) mpileup -EDS -C50 -m2 -F0.0005 -d 10,000’ and for BCFtools (v0.1.19) view ‘-p 0.99 -vcgN’. Functional consequences of the variants were produced by using the Ensembl VEP (McLaren *et al*, 2010).

### *MRE11* random mutagenesis

Plasmid pRS316 carrying *MRE11* coding sequence under the control of its natural promoter was transformed into mutagenic XL1-Red competent *E. coli* cells (Agilent Technologies) and propagated following the manufacturer's instructions. A plasmid library of ∽3,000 independent random mutant clones was transformed into *mre11*Δ*sae2*Δ cells, and transformants were screened for their ability to survive in the presence of camptothecin. Plasmids extracted from survivors loosing their camptothecin resistance after a passage on 5-fluoro-orotic acid (FOA) were sequenced and independently reintroduced in a *mre11*Δ*sae2*Δ strain.

### Molecular modelling

A monomeric molecular model of *S. cerevisiae* Mre11 was generated with the homology modelling program MODELLER (Sali & Blundell, 1993) v9.11, using multiple structures of Mre11 from *S. pombe* (PDB codes: 4FBW and 4FBK) and human (PDB code: 3T1I) as templates. A structural alignment of them was made with the program BATON (Sali & Blundell, 1990) and manually edited to remove unmatched regions. The quality of the model was found to be native-like as evaluated by MODELLER's NDOPE (−1.2) and GA341 (1.0) metrics and the QMEAN server (Benkert *et al*, 2009) (http://swissmodel.expasy.org/qmean/) (0.705). The monomeric model was subsequently aligned on the dimeric assembly of the 4FBW template to generate a dimer, and the approximate position of DNA binding was determined by aligning the *P. furiosus* structure containing dsDNA (PDB code: 3DSC) with the dimeric model. All images were obtained using the PyMOL Molecular Graphics System.

### Microscopy

Exponentially growing yeast strains carrying wild-type or mutant Mre11-YFP were treated with 40 Gy of ionising radiations with a Faxitron irradiator (CellRad). At regular intervals, samples were taken and fixed with 500 μl of Fixing Solution (4% paraformaldehyde, 3.4% sucrose). Cells were subsequently washed with wash solution (100 mM potassium phosphate pH 7.5, 1.2 M sorbitol) and mounted on glass slides. Images were taken at a DeltaVision microscope. All these experiments were carried out at 30°C.

### *In vitro* assays

For the electrophoretic mobility shift assay (EMSA), a radiolabelled DNA substrate (5 nM) was incubated with the indicated amount of Mre11 or Mre11^H37R^ in 10 μl buffer (25 mM Tris–HCl, pH 7.5, 1 mM DTT, 100 μg/ml BSA, 150 mM KCl) at 30°C for 10 min. The reaction mixtures were resolved in a 10% polyacrylamide gel in TBE buffer (89 mM Tris–borate, pH 8.0, 2 mM EDTA). The gel was dried onto Whatman DE81 paper and then subjected to phosphorimaging analysis. For nuclease assay, 1 mM MnCl_2_ was added to the reactions and the reaction mixtures were incubated at 30°C for 20 min and deproteinised by treatment with 0.5% SDS and 0.5 mg/ml proteinase K for 5 min at 37°C before analysis in a 10% polyacrylamide gel electrophoresis in TBE buffer.

Additional Materials and Methods can be found in the Supplementary Methods.

## Supporting Information

Supplementary Legends

Review Process File
