# Supplementary material for: Synthetic viability genomic screening defines Sae2 function in DNA repair
Source: EMBO J. 2015 Apr 21;34(11):1509–22. doi: 10.15252/embj.201590973 (PMC4474527; doi:10.15252/embj.201590973)
Supplement: Supplementary file 1 [file embj0034-1509-sd1.pdf]

Figure S1

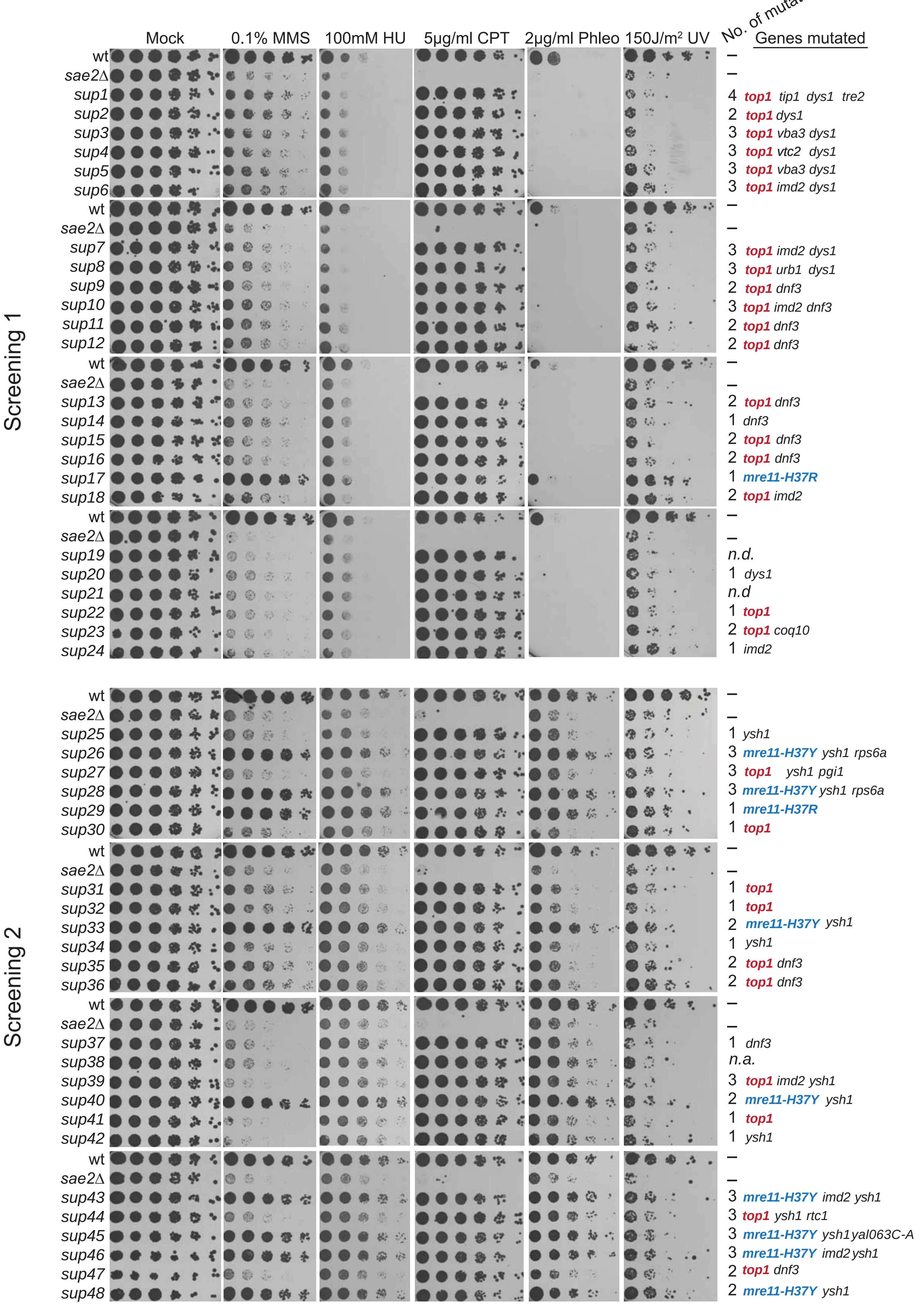

Mock
0.1% MMS
100mM HU
5μg/ml CPT
2μg/ml Phleo
150J/m² UV
No. of mutations
Genes mutated
Screening 1
wt –
sae2Δ –
sup1 4 top1 tip1 dys1 tre2
sup2 2 top1 dys1
sup3 3 top1 vba3 dys1
sup4 3 top1 vtc2 dys1
sup5 3 top1 vba3 dys1
sup6 3 top1 imd2 dys1
wt –
sae2Δ –
sup7 3 top1 imd2 dys1
sup8 3 top1 urb1 dys1
sup9 2 top1 dnf3
sup10 3 top1 imd2 dnf3
sup11 2 top1 dnf3
sup12 2 top1 dnf3
wt –
sae2Δ –
sup13 2 top1 dnf3
sup14 1 dnf3
sup15 2 top1 dnf3
sup16 2 top1 dnf3
sup17 1 mre11-H37R
sup18 2 top1 imd2
wt –
sae2Δ –
sup19 n.d.
sup20 1 dys1
sup21 n.d
sup22 1 top1
sup23 2 top1 coq10
sup24 1 imd2
Screening 2
wt –
sae2Δ –
sup25 1 ysh1
sup26 3 mre11-H37Y ysh1 rps6a
sup27 3 top1 ysh1 pgi1
sup28 3 mre11-H37Y ysh1 rps6a
sup29 1 mre11-H37R
sup30 1 top1
wt –
sae2Δ –
sup31 1 top1
sup32 1 top1
sup33 2 mre11-H37Y ysh1
sup34 1 ysh1
sup35 2 top1 dnf3
sup36 2 top1 dnf3
wt –
sae2Δ –
sup37 1 dnf3
sup38 n.a.
sup39 3 top1 imd2 ysh1
sup40 2 mre11-H37Y ysh1
sup41 1 top1
sup42 1 ysh1
wt –
sae2Δ –
sup43 3 mre11-H37Y imd2 ysh1
sup44 3 top1 ysh1 rtc1
sup45 3 mre11-H37Y ysh1 yal063C-A
sup46 3 mre11-H37Y imd2 ysh1
sup47 2 top1 dnf3
sup48 2 mre11-H37Y ysh1
