# Supplementary figures and images for: Synthetic viability genomic screening defines Sae2 function in DNA repair
Source: EMBO J. 2015 Apr 21;34(11):1509–22. doi: 10.15252/embj.201590973 (PMC4474527; doi:10.15252/embj.201590973)

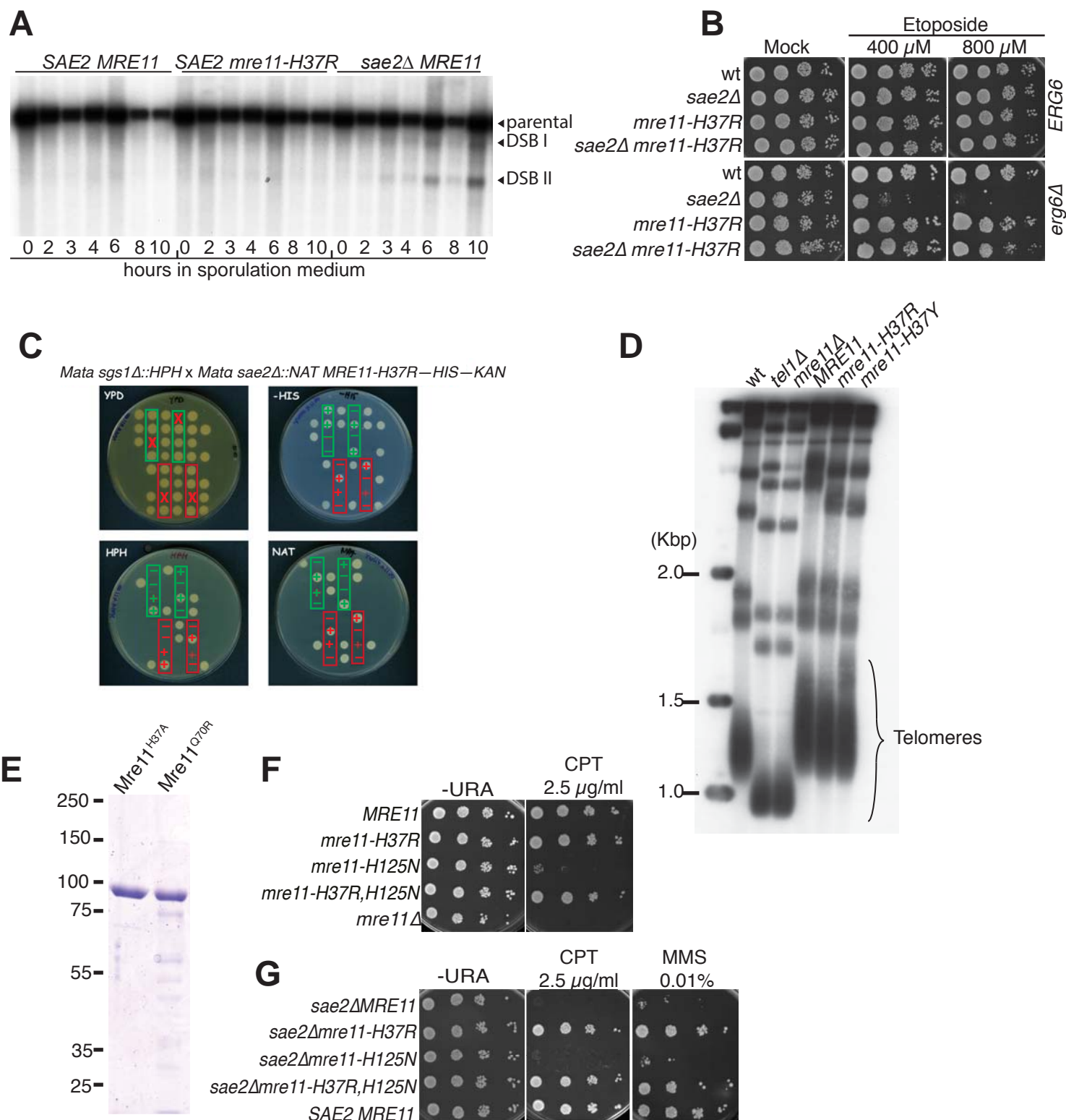

Supplement: Supplementary file 2 [file embj0034-1509-sd2.pdf]

**A**

| Mutation           | Times found |
|--------------------|-------------|
| <i>H37R</i>        | 1           |
| <i>P110L</i>       | 7           |
| <i>L89V</i>        | 1           |
| <i>P110L+I153L</i> | 1           |
| <i>P110L+S644G</i> | 1           |
| <i>Q70R+G193S</i>  | 1           |

**B**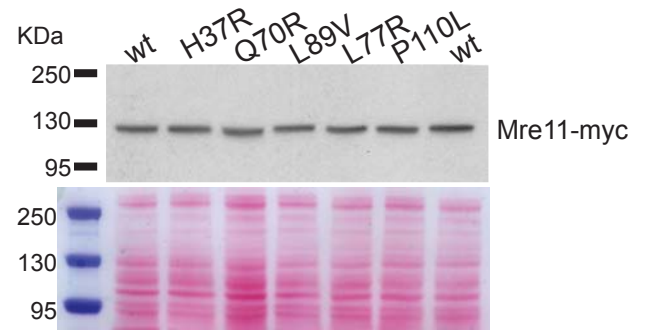**C**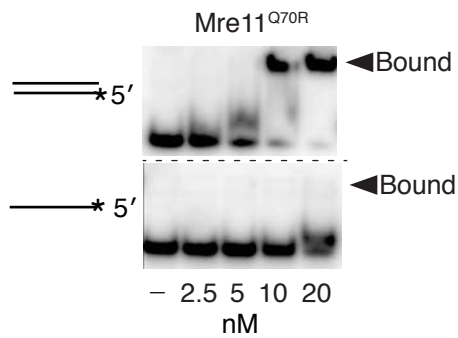**D**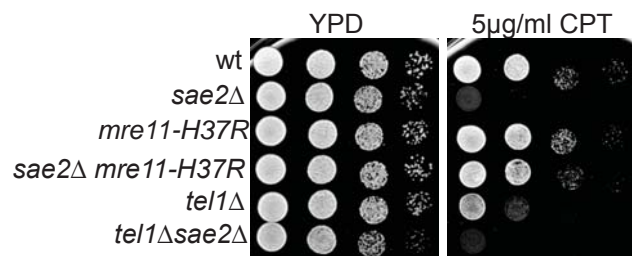

Supplement: Supplementary file 3 [file embj0034-1509-sd3.pdf]
