# Supplementary material for: Synthetic viability genomic screening defines Sae2 function in DNA repair
Source: EMBO J. 2015 Apr 21;34(11):1509–22. doi: 10.15252/embj.201590973 (PMC4474527; doi:10.15252/embj.201590973)
Supplement: Supplementary file 4 [file embj0034-1509-sd4.docx]

**Table S1: Yeast strains used in this study**

| **Name** | **Origin** | **Genotype** | **Backg.** |
| --- | --- | --- | --- |
| YTO1038 | This Study | *MATa/MATalpha mre11::MRE11-HIS3-KanMX6/mre11::MRE11-HIS3-KanMX6* | SK1 |
| YTO1039 | This Study | *MATa/MATalpha mre11::MRE11(H37R)-HIS3-KanMX6/mre11::MRE11(H37R)-HIS3-KanMX6* | SK1 |
| YTO1041 | This Study | *MATa/MATalpha sae2::NatMX6/sae2::NatMX6 mre11::MRE11-HIS3::KanMX6/mre11::MRE11-HIS3::KanMX6* | SK1 |
| YTO1042 | This Study | *MATa/MATalpha sae2::NatMX6/sae2::NatMX6 mre11::MRE11(H37R)-HIS3::KanMX6/ mre11::MRE11(H37R)-HIS3::KanMX6* | SK1 |
| YTO1000 | This Study | *MATa sae2::NatMX mre11::MRE11-HIS3-KanMX* | W303 |
| YTO1001 | This Study | *MATa sae2::NatMX mre11::mre11(H37R)-HIS3-KanMX* | W303 |
| YTO1002 | This Study | *MATa sae2::NatMX mre11::mre11(H37Y)-HIS3::KanMX* | W303 |
| YTO1003 | This Study | *MATa sae2::KanMX exo1::LEU2* | W303 |
| YTO1004 | This Study | *MATa sae2::KanMX mre11-H37Y* (suppressor No. 28) | W303 |
| YTO1005 | This Study | *MATa sae2::KanMX mre11-H37R* (suppressor No. 29) | W303 |
| YTO1028 | This Study | *MATa mre11::MRE11-HIS3-KanMX6* | W303 |
| YNG1/5c | This Study | *MATa mre11::mre11(H37Y)-HIS3-KanMX6 xrs2∆::LEU2* | W303 |
| DDY026 | This Study | *MATa xrs2::KanMX4* | W303 |
| YLL125B | This Study | *MATa mre11::MRE11-HIS3-KANMX6 rad50S::LEU2* | W303 |
| YLL126 | This Study | *MATa mre11::mre11(H37R)-HIS3-KANMX6 rad50S::LEU2* | W303 |
| YTO1029 | This Study | *MATa mre11::mre11(H37R)-HIS3-KanMX6* | W303 |
| YTO1030 | This Study | *MATa mre11::mre11(H37Y)-HIS3::KanMX6* | W303 |
| YTO1031 | This Study | *MATalpha sae2::NatMX mre11::MRE11-HIS3-KanMX* | W303 |
| YTO1032 | This Study | *MATalpha sae2::NatMX mre11::mre11(H37R)-HIS3-KanMX* | W303 |
| YTO1033 | This Study | *MATalpha sae2::NatMX yku70::LEU2 mre11::MRE11-HIS3::KanMX* | W303 |
| YTO1034 | This Study | *MATalpha sae2::NatMX yku70::LEU2 mre11::MRE11(H37R)-HIS3::KanMX* | W303 |
| YTO1035 | This Study | *MATalpha sae2::NatMX yku70::LEU2 mre11::MRE11(H37Y)-HIS3::KanMX* | W303 |
| YLL135 | This study | *MATa sae2::sae2-2,5,6,8,9-LEU2-KanMX6 mre11::MRE11-HIS3-HPH* | W303 |
| YLL136 | This study | *MATa sae2::sae2-2,5,6,8,9-LEU2-KanMX6 mre11::mre11(H37Y) -HIS3-HPH* | W303 |
| YFP1177/2d | This study | *MATa MRE11-LLAKKRKG-YFP* | W303 |
| YFP1178/5b | This study | *MATa MRE11-LLAKKRKG-YFP sae2::KanMX6* | W303 |
| YFP1193/3d | This study | *MATa mre11(H37R)-LLAKKRKG-YFP* | W303 |
| YFP1185 | This study | *MATa mre11(H37R)-LLAKKRKG-YFP sae2::KanMX6* | W303 |
| YFP1216/1b | This study | *MATa MRE11-LLAKKRKG-YFP rad50S-LEU2* | W303 |
| YFP1217/4b | This study | *MATa mre11(H37R)-LLAKKRKG-YFP rad50S-LEU2* | W303 |
| YFP1213 | This study | *MATa MRE11-LLAKKRKG-YFP rad51::HIS3* | W303 |
| YFP1219/7c | This study | *MATa MRE11-LLAKKRKG-YFP rad51::HIS3 sae2::NatMX6* | W303 |
| YFP1242/2d | This study | *MATa mre11(H37R)-LLAKKRKG-YFP rad51::HIS3 sae2::NatMX6* | W303 |
| YLL116 | This study | *MATa mec1::TRP1 sml1::LEU2 mre11::MRE11-HIS3-KanMX6* | W303 |
| YLL117/5c | This study | *MATa mec1::TRP1 sml1::LEU2 mre11::mre11(H37R)-HIS3-KanMX6* | W303 |
| YLL116/8b | This study | *MATa mec1::TRP1 sml1::LEU2 sae2::NAT mre11::MRE11-HIS3-KanMX6* | W303 |
| YLL117/7d | This study | *MATalpha mec1::TRP1 sml1::LEU2 sae2::NAT mre11::mre11(H37R)-HIS3-KanMX6* | W303 |
| YMV80 | J. Haber | *MATa∆::hisG his4::NatMX-leu2-(XhoI to D718)* *hmr∆::ADE1* *hml∆::ADE1* *ade3::GAL::HO* *ura3-52* *leu2-cs* *lys5* | YMV80 |
| YTO1077 | This study | *sae2::KanMX mre11::MRE11-TRP1::hphMX* | YMV80 |
| YTO1078 | This study | *sae2::KanMX mre11::MRE11(H37R)-TRP1::hphMX* | YMV80 |
| YFP1259/5d | This study | *MATa MRE11-LLAKKRKG-YFP sae2::KanMX6 tel1∆::HIS3* | W303 |
| YFP1265/2c | This study | *MATa mre11(H37R)-LLAKKRKG-YFP sae2::KanMX6 tel1∆::HIS3* | W303 |
| YLL127 | This study | *MATa mre11::MRE11-HIS3-KanMX6 tel1::LEU2* | W303 |
| YLL128 | This study | *MATa mre11::MRE11-HIS3-KanMX6 tel1::LEU2 sae2::NAT* | W303 |
| YLL129 | This study | *MATa mre11::mre11(H37R)-HIS-KanMX6 tel1::LEU2* | W303 |
| YLL130 | This study | *MATa mre11::mre11(H37R)-HIS-KanMX6 tel1::LEU2 sae2::NAT* | W303 |
| YFP1305/1C | This study | *MATalpha erg6∆::TRP1* | W303 |
| YFP1306/1A | This study | *MATa erg6∆::TRP1 sae2∆::NatMX6* | W303 |
| YFP1307/2D | This study | *MATa erg6∆::TRP1 mre11(H37R) –HisMX6-KanMX6* | W303 |
| YFP1308/6A | This study | *MATa erg6∆::TRP1 sae2∆::NatMX6 mre11(H37R) –HisMX6-KanMX6* | W303 |

**W303** is *ade2-1 ura3-1 his3-11,15 trp1-1 leu2-3112 can1-100 RAD5*

**SK1** is *ho::LYS2 ura3 leu2::hisG trp1::hisG his3::hisG*
