# Supplementary material for: Synthetic viability genomic screening defines Sae2 function in DNA repair
Source: EMBO J. 2015 Apr 21;34(11):1509–22. doi: 10.15252/embj.201590973 (PMC4474527; doi:10.15252/embj.201590973)
Supplement: Supplementary file 5 [file embj0034-1509-sd5.docx]

**Table S2: Plasmids used in this study**

| **Plasmid name** | **Description** |
| --- | --- |
| P1660/pIG20 | pRS303-*MRE11* |
| P1661 | pRS303*-mre11(H37R)* |
| P1664 | pRS303*-mre11(H37Y)* |
| P1673 | pRS303*-mre11(H125N)* |
| P1674 | pRS303*-mre11(H37R,H125N)* |
| P1678 | pRS303*-mre11(H37A)* |
| P1685 | pRS416*-MRE11-13myc* |
| P1662 | pRS416*-mre11-H37R-13myc* |
| P1688 | pRS416-*mre11-H125N-13myc* |
| P1690 | pRS416*-mre11(H37R,H125N)-13myc* |
| pFP119 | pRS416*-mre11(P110L)-13myc* |
| pFP120 | pRS416*-mre11(L89V)-13myc* |
| pFP121 | pRS416*-mre11(Q70R)-13myc* |
| pFP125 | pRS416*-mre11(L77R)-13myc* |
| pFP118.1 | pRS306*-mre11(H37R)-∆CT* |
