# Supplementary material for: Synthetic viability genomic screening defines Sae2 function in DNA repair
Source: EMBO J. 2015 Apr 21;34(11):1509–22. doi: 10.15252/embj.201590973 (PMC4474527; doi:10.15252/embj.201590973)
Supplement: Supplementary file 6 [file embj0034-1509-sd6.docx]

**Table S3. Whole-genome sequencing coverage by sample**

| Suppr. | Raw (Gbp) | Mapped (Gbp) | Mapped-dups (Gbp) | Estim. Coverage (fold) |
| --- | --- | --- | --- | --- |
| 1 | 0.72 | 0.68 | 0.68 | 48.57 |
| 2 | 0.65 | 0.61 | 0.6 | 42.86 |
| 3 | 0.68 | 0.64 | 0.63 | 45 |
| 4 | 0.65 | 0.61 | 0.61 | 43.57 |
| 5 | 0.59 | 0.55 | 0.54 | 38.57 |
| 6 | 0.63 | 0.57 | 0.56 | 40 |
| 7 | 0.64 | 0.58 | 0.58 | 41.43 |
| 8 | 0.72 | 0.66 | 0.65 | 46.43 |
| 9 | 0.67 | 0.62 | 0.61 | 43.57 |
| 10 | 0.6 | 0.56 | 0.56 | 40 |
| 11 | 0.59 | 0.54 | 0.54 | 38.57 |
| 12 | 0.57 | 0.54 | 0.52 | 37.14 |
| 13 | 0.66 | 0.61 | 0.61 | 43.57 |
| 14 | 0.58 | 0.54 | 0.54 | 38.57 |
| 15 | 0.64 | 0.59 | 0.59 | 42.14 |
| 16 | 0.63 | 0.57 | 0.57 | 40.71 |
| 17 | 0.7 | 0.64 | 0.63 | 45 |
| 18 | 0.66 | 0.6 | 0.59 | 42.14 |
| 19 | 0.68 | 0.61 | 0.61 | 43.57 |
| 20 | 0.64 | 0.6 | 0.59 | 42.14 |
| 21 | 0.65 | 0.61 | 0.61 | 43.57 |
| 22 | 0.68 | 0.63 | 0.62 | 44.29 |
| 23 | 0.72 | 0.67 | 0.66 | 47.14 |
| 24 | 0.74 | 0.68 | 0.67 | 47.86 |
| 25 | 0.73 | 0.68 | 0.67 | 47.86 |
| 26 | 0.73 | 0.7 | 0.7 | 50 |
| 27 | 0.7 | 0.68 | 0.67 | 47.86 |
| 28 | 0.72 | 0.7 | 0.69 | 49.29 |
| 29 | 0.72 | 0.68 | 0.68 | 48.57 |
| 30 | 0.71 | 0.68 | 0.66 | 47.14 |
| 31 | 0.68 | 0.64 | 0.64 | 45.71 |
| 32 | 0.68 | 0.64 | 0.63 | 45 |
| 33 | 0.68 | 0.65 | 0.65 | 46.43 |
| 34 | 0.68 | 0.65 | 0.65 | 46.43 |
| 35 | 0.72 | 0.67 | 0.66 | 47.14 |
| 36 | 0.61 | 0.58 | 0.57 | 40.71 |
| 37 | 0.6 | 0.58 | 0.56 | 40 |
| 39 | 0.61 | 0.58 | 0.57 | 40.71 |
| 40 | 0.58 | 0.56 | 0.54 | 38.57 |
| 41 | 0.65 | 0.63 | 0.61 | 43.57 |
| 42 | 0.64 | 0.61 | 0.61 | 43.57 |
| 43 | 0.63 | 0.59 | 0.59 | 42.14 |
| 44 | 0.67 | 0.63 | 0.63 | 45 |
| 45 | 0.59 | 0.57 | 0.57 | 40.71 |
| 46 | 0.61 | 0.58 | 0.58 | 41.43 |
| 47 | 0.54 | 0.52 | 0.51 | 36.43 |
| 48 | 0.54 | 0.52 | 0.52 | 37.14 |
| sae2∆_1 | 0.59 | 0.54 | 0.54 | 38.57 |
| sae2∆_2 | 0.57 | 0.53 | 0.52 | 37.14 |
| sae2∆_3 | 0.7 | 0.65 | 0.64 | 45.71 |
