# Supplementary material for: Synthetic viability genomic screening defines Sae2 function in DNA repair
Source: EMBO J. 2015 Apr 21;34(11):1509–22. doi: 10.15252/embj.201590973 (PMC4474527; doi:10.15252/embj.201590973)
Supplement: Supplementary file 7 [file embj0034-1509-sd7.docx]

**Supplementary Methods**

**Western Blot.** Exponentially growing cells were treated with appropriate DNA damaging agents (CPT 5 µg/ml, Phleomycin 3 µg/ml) and proteins were extracted in the presence of trichloroacetic acid (TCA). Proteins were separated on 10% acrylamide gels and transferred to nitrocellulose membranes. Anti-Rad53 antibodies (Abcam ab104232) were used to measure checkpoint activation.

**Protein expression and purification** To express Mre11 and Mre11^H37R^ proteins, the open reading frame of *MRE11* was PCR amplified and inserted in the pESC-URA vector to fuse *MRE11* to a C-terminal FLAG tag. The *mre11-H37R* mutation was then introduced by site-directed mutagenesis. These plasmids were introduced into the protease deficient yeast strain BJ5464. An overnight culture was diluted 1:100 into 12 l of –Ura SD with 2% raffinose. Cells were cultured at 30°C until the OD660 reached 0.8, at which time galactose was added to 2% to induce Mre11 expression. Cells were harvested by centrifugation after 16 h of further incubation, and the pellet (~50 g) was stored frozen at -80°C. After grinding the yeast pellet with dry ice, 50 ml of ice-cold lysis buffer (40 mM KH2PO4, pH 7.4, 20% glycerol, 1 mM EDTA, 0.1% NP-40, 2 mM DTT, 200 mM KCl, and the cocktail of protease inhibitors consisting of aprotinin, chymostatin, leupeptin, and pepstatin A at 5 μg/ml each, and also 1 mM phenyl-methylsulfonyl fluoride was added to the extract. All the subsequent steps were carried out at 0-4°C. The extract was clarified by ultracentrifugation (100,000g for 45 min) and then applied onto a 5 ml SP Sepharose column. After washing the column with 100 ml K buffer (20 mM KH2PO4, pH 7.4, 10% glycerol, 0.5 mM EDTA, 0.01% Igepal, 1 mM DTT) plus 100 mM KCl, it was developed with a 100 ml linear gradient from 100 to 575 mM KCl. The peak fractions were pooled and incubated with 0.5 ml of anti-FLAG M2 agarose beads for 2 h. The matrix was washed once with 20 ml K buffer containing 500 mM KCl and 2 mM each of ATP and MgCl2, and then three additional times with 20 ml of K buffer containing 500 mM KCl. Mre11 or mre11-H37R was eluted with 1 ml of K buffer plus 500 mM KCl and 200 μg/ml FLAG peptide for 1 h. The eluate that contained purified Mre11 or Mre11-H37R was dialyzed against K buffer with 500 mM KCl overnight before being concentrated using an Ultracel-30K concentrator (Amicon) to 4 mg/ml and stored at -80°C in small aliquots.

**DNA substrates**: Oligo H1 (5’-TTGATAAGAGGTCATTTGAATTCATGGCTTAGAGC

TTAATTGCTGAATCTGGTGCTGGGATCCAACATGTTTTAAATATG-3’) was 3’ end labeled with [α-^32^P] Cordycepin 5ʹ-triphosphate (PerkinElmer) and terminal deoxytransferase (Roche) to use as single stranded DNA substrate. To make duplex DNA substrate for electrophoretic mobility shift and nuclease assays, radiolabeled H1 was annealed to H2 (5’ –CATATTTAAAACATGTTGGATCCCAGCA

CCAGATTCAGCAATTAAGCTCTAAGCCATGAATTCAAATGACCTCTTATCAA-3’).
