# Supplementary material for: Synthetic viability genomic screening defines Sae2 function in DNA repair
Source: EMBO J. 2015 Apr 21;34(11):1509–22. doi: 10.15252/embj.201590973 (PMC4474527; doi:10.15252/embj.201590973)
Supplement: Supplementary file 8 [file embj0034-1509-sd8.docx]

**SUPPLEMENTARY FIGURE LEGENDS**

**Figure S1 – Validation of the suppression phenotype for 48 suppressors recovered from the screen.**

Each strain was tested for resistance to MMS, HU, camptothecin (CPT), phleomycin (Phleo), or ultraviolet light (UV). Each strain is shown along with the mutations identified in it.

**Figure S2 – Additional phenotypes of the *mre11-H37R* mutant.**

A *mre11-H37R* is not a *mre11S*-like allele, as it does not display accumulation of unprocessed meiotic DSBs (as in a *sae2∆* strain) at the *THR4* locus.

B *mre11-H37R* suppresses *sae2∆* hypersensitivity to etoposide. 10-fold serial dilutions of overnight grown cultures of the indicated strains, either in an *ERG6* or *erg6∆* background were spotted on plates containing the indicated amounts of etoposide. Images were taken 2 to 3 days later.

C Result of a cross between an *sgs1∆* strain and a *sae2∆mre11-H37R* strain. In green are tetrads containing *sae2∆sgs1∆* double mutants; in red are tetrads containing *sae2∆sgs1∆mre11-H37R* triple mutants. (+: strain grew; –: strain did not grow; X: spore died after germination).

D Telomere length in the indicated strains was assessed by digesting genomic DNA with restriction enzyme XhoI, subjecting samples to agarose gel eletrophoresis and then assessing by hybridization with a telomere-specific probe.

E Mre11^H37A^ and Mre11^Q70R^ were purified from yeast cell extracts overexpressing the corresponding proteins.

F Intragenic suppression of camptothecin (CPT) hypersensitivity of *mre11-nd* (*mre11-H125N*) by *mre11-H37R*.

G Mre11 nuclease activity is not required for *mre11-H37R* mediated suppression of *sae2∆* CPT hypersensitivity*.*

**Figure S3 – Gene-directed random mutagenesis screen identifies additional *mre11^supsae2∆^* alleles.**

A Mutations found in the plasmids recovered from the screen for new *mre11^supsae2∆^* alleles. Numbers indicate the frequency of each allele.

B *mre11^supsae2∆^* alleles do not alter Mre11 protein levels: protein extracts from strains carrying a Myc-tagged version of the indicated *mre11* alleles were analysed by SDS-PAGE and western blot with the 9E10 antibody.

C Electrophoretic mobility shift assays on Mre11^Q70R^  with dsDNA or ssDNA.

D Deletion of *TEL1* does not suppress the CPT hypersensitivity of a *sae2∆* strain.

**SUPPLEMENTARY TABLE LEGENDS**

**Table S1 – Yeast strains used in this study**

**Table S2 – Plasmids used in this study**

**Table S3 – Whole genome sequencing coverage data by sample**.
