# Supplementary material for: Synthetic viability genomic screening defines Sae2 function in DNA repair
Source: EMBO J. 2015 Apr 21;34(11):1509–22. doi: 10.15252/embj.201590973 (PMC4474527; doi:10.15252/embj.201590973)
Supplement: Supplementary file 9 [file embj0034-1509-sd9.pdf]

THE
EMBO
JOURNAL

Manuscript EMBO-2015-90973

Synthetic-viability genomic screening defines Sae2 function in DNA repair

Fabio Puddu, Tobias Oelschlaegel, Ilaria Guerini, Nicola J. Geisler, Hengyao Niu, Mareike Herzog, Israel Salguero, Bernardo Ochoa-Montaño, Emmanuelle Viré, Patrick Sung, David J. Adams, Thomas M. Keane and Stephen P Jackson

Corresponding author: Stephen P Jackson, Wellcome Trust Cancer Research UK Institute

---

Review timeline:

Submission date:	08 January 2015
Editorial Decision:	30 January 2015
Revision received:	16 March 2015
Accepted:	02 April 2015

Editor: Hartmut Vodermaier

Transaction Report:

(Note: With the exception of the correction of typographical or spelling errors that could be a source of ambiguity, letters and reports are not edited. The original formatting of letters and referee reports may not be reflected in this compilation.)

1st Editorial Decision 30 January 2015

Thank you for submitting your manuscript on Sae2-Mre11 interplay to The EMBO Journal. It has now been reviewed by three expert referees, whose comments you will find copied below. Given their positive overall assessment, we would be happy to consider the study further for publication, pending satisfactory addressing of a number of concerns raised in their reports. As you will see, these issues mostly pertain to strengthening the evidence in support of the presented model, that suppression of Sae2 deletion phenotypes by specific Mre11 mutants primarily reflects an Sae2 role in removing Mre11 from DNA. Since these concerns are clearly explained in the referee reports, I will not repeat them in detail here but would be happy to discuss specific points further with you if needed.

At this stage, I would therefore like to invite you to prepare a revised manuscripts along the lines suggested by the referees, keeping in mind that we allow only a single round of major revision, and that it will therefore be important to carefully answer to all points raised at this stage. Please also note that we now require a completed 'author checklist' to be submitted with all revised manuscripts - see below for more details. A final editorial point regarding data presentation in graphs, relating to Figure 6A: please make sure to connect individual data points by straight and not curved lines.

© European Molecular Biology Organization

Referee #1:

The MRX and Sae2 proteins have multiple important functions in DNA damage response. Some of them are well defined but a lot remains unclear. Puddu and colleagues carried out genome-wide analysis to look for suppressors of DNA-damage sensitivity associated with sae2 deletion. These studies are important, as they can identify genetic interactions and explain individual functions. The authors identified mutations in the N-terminal region of Mre11. These mutations rescue sensitivity of sae2delta not only to camptothecin but also to other drugs, and thus reflect a general function of the complex. It is shown that not all phenotypes of sae2delta are rescued by the Mre11 mutations. Interestingly, the Mre11 mutations rescue even in the absence of Mre11 nuclease activity, and thus the Mre11-h37r phenotype cannot be explained by its nuclease being uncoupled from Sae2. Rather, the authors show that the mutated Mre11 has reduced capacity to bind ssDNA, and propose a model where the wild type MRX complex must be removed by Sae2 from the DNA end. In the absence of Sae2, wt MRX stuck at the DNA end can block HR. The mutated MRX complex does not need this function of Sae2 because it has a lower intrinsic ssDNA binding capacity. This is supported by IF data showing that indeed Mre11-h37r persists less at DNA damage sites in sae2delta.

The study is well done and presented. I only have minor (and mainly discussion) points.

1. End of introduction and beginning of discussion: please explain specifically which "apparent paradoxes" are addressed.

2. Both mre11-nd and sae2 delta mutants (sae2 delta more than mre11-nd) are sensitive to CPT and IR (as also mentioned in the introduction). It is demonstrated that the rescue due to mutations in Mre11 occurs even in the absence of mre11 nuclease activity (that is, in mre11-nd background). Can you provide quantitation of these data? I would expect that the Mre11 mutation will rescue sae2 delta up to mre11-nd level, but not to wt level. Is that correct? This would fit with the model later where it is discussed that Sae2 has two functions that affect CPT sensitivity: to promote Mre11 nuclease and remove Mre11 from ends.

3. Fig. 2B: how was the western blot done? Mre11 was tagged or was anti-Mre11 antibody used?

4. Page 12: Has it been demonstrated that MRX complex bound at ssDNA end indeed "delays downstream HR events?" I believe this is only a model and should be discussed as such.

5. Figure 6A: What is the kinetics of mre11-nd? Is it similar to sae2delta mre11-h37r? The author's model would predict this. The persistence of Mre11 at DNA damage sites might therefore be again regulated by its capacity (a) to cut DNA and (b) to be actively removed, both of these functions regulated by Sae2. Is it correct?

5. Deletions of SGS1 and SAE2 result in synthetic sick/lethal phenotype, possibly due to telomeric functions. Have the authors looked whether the Mre11 mutations rescue the slow growth of sae2 sgs1?

Referee #2:

The manuscript reports an identification of a suppressor, mre11-H37R that improves the viability of sae2 deletion on camptothecin and other genotoxic agent treatment. Analysis of mre11-H37R and other mre11 variants selected for their ability to suppress the lethality of sae2 deletion to DNA damaging agents suggests that one of the key functions of Sae2 in recombination is to remove Mre11 complex from DNA ends with single stranded DNA. A redundant role of Tel1 in the Mre11 removal from DNA ends was suggested based on the observation that the suppressive function of mre11-H37R on sae2 deletion depends on Tel1 protein.
The manuscript is well composed and the results are convincing. The idea that Sae2 is involved in the Mre11 complex dissociation was suggested for a while ago (see Lisby et al, 2004) but has not been tested comprehensively as they did and thus is novel. Inclusion of biochemical, genetic and structural analyses further offer more complete picture of this process. I have a couple of questions

and suggestions that require addressing experimentally and through rewriting the manuscript to clarify a few points.

1. Evidence suggests that removal of Mre11 complex from DNA ends could be coupled to resection event itself. For instance, sgs1-D664delta is deficient in resection and synthetic lethal to sae2 deletion, causing Mre11 to persist at DNA ends (Genetics. 2013 195(4):1241-51). The one model is thus that by virtue of endonuclease activity, Mre11 liberates itself from DNA and Sae2 could simply promote endonuclease activity of Mre11 to assist this process. In this scenario, one anticipates that mre11-H37R should bypass the requirement of Sae2 in endonucleolytic removal of Mre11 at DNA ends.

2. It is certainly logical that mre11-H37R could suppress sae2 deletion due to a reduced affinity to ssDNA and thereby becomes more prone to dissociate from DNA ends. Biochemical results support this idea. However, one cannot exclude the possibility that the mutation allows the endonuclease activity of other nuclease especially, Dna2, to gain access to and cleave DNA ends to substitute Mre11 endonuclease activity in sae2 deletion. The effect of DNA2 deletion on the CPT sensitivity of mre11-H37R sae2 should be tested.

3. Furthermore, resection in sae2 mre11-H37R exo1 should be examined in yeast strain following an induction of non-repairable HO break to test if the initial resection is improved by the mre11-H37R mutation.

4. In page 13, "mre11-nd cells are only impaired in Mre11 nuclease activity" I do not recall any studies testing the Mre11 level at DNA ends in mre11-H125N or tel1 mutants. It should be tested to support this hypothesis.

5. The reference listed as "Chen et al" is without Journal information and could not be found from pubmed search.

Referee #3:

This study by Puddu and Jackson et al. examines the role of the Sae2 protein in budding yeast and its relationship to the Mre11-Rad50-Xrs2 (MRX) complex in DNA repair. Sae2 null mutants are deficient in meiotic double-strand break repair and also show sensitivity to DNA damaging agents in mitotic cells. Here the authors select for genetic suppressors of the sae2 phenotype in yeast and identify a number of alleles in various genes that can nearly completely suppress the camptothecin sensitivity of a sae2 strain. Remarkably, one particular residue of the Mre11 protein is found to be the site of a missense mutation in several of these strains, and the authors show that this allele acts as a recessive suppressor of some of the mitotic phenotypes of sae2, but not its meiotic phenotype. In vitro analysis indicates that this mutant is not hyperactive for nuclease activity as one might expect, but appears to be partially deficient in DNA binding. This overall result coincides with a previous publication showing that sae2 strains retain the MRX complex for a longer time and at higher levels than a wild-type strain. In addition, the nearly complete suppression of sae2 camptothecin sensitivity by various alleles of Mre11 suggest that a significant component of the sae2 phenotype may be attributed to inappropriate MRX binding to DNA.

This is an important study that is likely to be of interest to many in the DNA damage response field. The data and presentation is generally sound, although I do not think there is sufficient data here to conclusively say what is the exact defect in Mre11 that is exhibited by the suppressor mutants. It could be a DNA-binding defect, although complete evidence for this is lacking. The model presented suggests that this is the foregone conclusion here, but better evidence for this as well as some other validation of the existing experiments is also necessary.

1. It is interesting that the H37Y/R suppressor mutations in Mre11 do not suppress the meiotic defect in sae2 strains. Since the protein-DNA conjugate in the case of Spo11 would be a 5' adduct, in contrast to the 3' TOP1 adduct with camptothecin, the authors should examine whether the mre11 suppressor allele works when sae2 strains are tested for survival on a topoisomerase II poison such as etoposide.

2. Since the H37 residue is highly conserved and is in a region predicted to contact DNA, as the authors point out, it seems unlikely that the H37A mutant is completely normal for DNA binding while the H37R and H37Y mutants are not. This should be explicitly tested in vitro with purified proteins and in the Mre11 foci assay to determine if loss of binding affinity is actually the reason for the H37R/Y suppression of sae2 phenotypes.

3. The same issue also arises with the Q70R, L89V, and P110L mutants - are these all deficient in DNA binding in vitro? Do they show a specific defect in single-stranded DNA binding? Is there a preference for the polarity of single-stranded DNA binding? Also, the levels of these proteins in vivo should be confirmed as it was for the H37 mutants.

4. It is interesting that the sup28 and sup29 alleles of mre11 are recessive for suppression of the CPT phenotype of a sae2 strain (Fig. 2C). One would think that the same scenario is occurring in the presence of other types of damage, but it appears that the alleles are not recessive when measured for survival to MMS. Why is this?

5. The message here seems to be that the sae2 damage sensitivity phenotype is primarily reflecting the block to DSB repair imposed by improperly bound MRX, and not from a deficiency in resection alone. If this is the case, why is SSA still impaired in the sae2 strain and is not recovered by mre11-H37R? Presumably this is not a resection defect? If so then what is the defect in a sae2 strain undergoing SSA?

6. Fig. 4: Mre11 does not bind to DNA on its own - it is part of the larger complex with Rad50 and Xrs2. Are the H37R mutant and the other mutants forming the MRX complex normally in vivo? What is the DNA binding capacity of H37R in the form of MRX?

7. The fact that the H37R allele of Mre11 suppresses the camptothecin sensitivity of the Rad50S allele suggests that Rad50S versions of MRX may bind to single-stranded DNA with higher affinity or longevity compared to wild-type Rad50; is this the case?

8. It has been shown that in S.pombe, 5'-adduct removal depends on both Rad32(Mre11) and Ctp1(Sae2) while the removal of 3' adducts requires only Rad32. (http://www.ncbi.nlm.nih.gov/pubmed/19150433) It would be informative here if the authors could explain how the current study fits into these existing data.

1st Revision - authors' response 16 March 2015

Referee #1:

The MRX and Sae2 proteins have multiple important functions in DNA damage response. Some of them are well defined but a lot remains unclear. Puddu and colleagues carried out genome-wide analysis to look for suppressors of DNA-damage sensitivity associated with sae2 deletion. These studies are important, as they can identify genetic interactions and explain individual functions. The authors identified mutations in the N-terminal region of Mre11. These mutations rescue sensitivity of sae2delta not only to camptothecin but also to other drugs, and thus reflect a general function of the complex. It is shown that not all phenotypes of sae2delta are rescued by the Mre11 mutations. Interestingly, the Mre11 mutations rescue even in the absence of Mre11 nuclease activity, and thus the Mre11-h37r phenotype cannot be explained by its nuclease being uncoupled from Sae2. Rather, the authors show that the mutated Mre11 has reduced capacity to bind ssDNA, and propose a model where the wild-type MRX complex must be removed by Sae2 from the DNA end. In the absence of Sae2, wt MRX stuck at the DNA end can block HR. The mutated MRX complex does not need this function of Sae2 because it has a lower intrinsic ssDNA binding capacity. This is supported by IF data showing that indeed Mre11-h37r persists less at DNA damage sites in sae2delta.

The study is well done and presented. I only have minor (and mainly discussion) points.

1. End of introduction and beginning of discussion: please explain specifically which "apparent paradoxes" are addressed.

We have revised our text to explain what we mean by “apparent paradoxes”. Please see our revised text towards the bottom of page 4.

2. Both mre11-nd and sae2 delta mutants (sae2 delta more than mre11-nd) are sensitive to CPT and IR (as also mentioned in the introduction). It is demonstrated that the rescue due to mutations in Mre11 occurs even in the absence of mre11 nuclease activity (that is, in mre11-nd background). Can you provide quantitation of these data? I would expect that the Mre11 mutation will rescue sae2 delta up to mre11-nd level, but not to wt level. Is that correct? This would fit with the model later where it is discussed that Sae2 has two functions that affect CPT sensitivity: to promote Mre11 nuclease and remove Mre11 from ends.

We thank Referee 1 for raising these issues. We have now quantified these data. As shown in figure 4F, the *mre11-H37R* mutation does not rescue *sae2*Δ up to *mre11-H125N* levels but to a higher level. We feel that this was expected, given that *mre11H37R* can also suppress the *mre11H125N* mutation. Interestingly, the sensitivities of *sae2*Δ*mre11H37R* and *mre11H125N, H37R* strains are comparable. This is in line with a model in which the H37R mutation suppresses lack of Mre11 nuclease activity either caused by loss of Sae2 or by lack of the proper residue in the Mre11 active site. While it is known that Mre11 nuclease activity is required for the removal of Mre11 from DSB ends, the fact that the double mutant *sae2*Δ*mre11H125N* is more sensitive than *mre11H125N,* but epistatic with *sae2*Δ (see figures 4E and 4F) suggests that Sae2 has some other function apart from activating Mre11 nuclease activity. We conclude that this second function is connected with the removal of Mre11, because *mre11-H37R* can suppress both the lack of nuclease activity and the second function of Sae2 (*mre11H37R,H125N* and *sae2*Δ*mre11H37R* strains display similar DNA-damage sensitivities, despite a *sae2*Δ strain being more sensitive than a *mre11H125N* strain; the four strains depicted in figure 4F).

3. Fig. 2B: how was the western blot done? Mre11 was tagged or was anti-Mre11 antibody used?

The western blot in figure 2B was carried out using anti-Mre11 antibodies, as now specified in the legend to the figure.

4. Page 12: Has it been demonstrated that MRX complex bound at ssDNA end indeed "delays downstream HR events?" I believe this is only a model and should be discussed as such.

The reviewer correctly notes that it has never been demonstrated before that persistent binding of Mre11 to the 3’ end hinders HR. We have reworded the sentence to reflect this fact (2/3 down page 13).

5. Figure 6A: What is the kinetics of mre11-nd? Is it similar to sae2delta mre11-h37r? The author's model would predict this. The persistence of Mre11 at DNA damage sites might therefore be again regulated by its capacity (a) to cut DNA and (b) to be actively removed, both of these functions regulated by Sae2. Is it correct?

In regard to these points Lisby et al. (Lisby *et al*, 2004) showed that *mre11-nd* strains (H125N, or D56A) displayed increased persistence of Mre11 at DSB ends, a phenotype that was comparable to but less strong than that of a *sae2*Δ strain. We show that *mre11-H37R* does not completely rescue the dissociation of Mre11 from foci in a *sae2*Δ strain (the kinetics of the double mutant are intermediate between those of wild-type and *sae2*Δ strains). It is therefore likely that *mre11-H125N* and *sae2mre11H37R* may have a similar kinetics of persistence at DNA-damage sites.

5. Deletions of SGS1 and SAE2 result in synthetic sick/lethal phenotype, possibly due to telomeric functions. Have the authors looked whether the Mre11 mutations rescue the slow growth of sae2 sgs1?

Following the suggestion of this referee, we checked whether *mre11-H37R* could suppress the synthetic lethality/sickness of a *sgs1*Δ*sae2*Δ strain. As detailed in the new Figure E2C, this is not the case. Additionally, we show in the new Figure E2D that the *mre11-H37R* strain is apparently fully

proficient for telomere length maintenance.

Referee #2:

The manuscript reports an identification of a suppressor, mre11-H37R that improves the viability of sae2 deletion on camptothecin and other genotoxic agent treatment. Analysis of mre11-H37R and other mre11 variants selected for their ability to suppress the lethality of sae2 deletion to DNA damaging agents suggests that one of the key functions of Sae2 in recombination is to remove Mre11 complex from DNA ends with single stranded DNA. A redundant role of Tel1 in the Mre11 removal from DNA ends was suggested based on the observation that the suppressive function of mre11-H37R on sae2 deletion depends on Tel1 protein. The manuscript is well composed and the results are convincing. The idea that Sae2 is involved in the Mre11 complex dissociation was suggested for a while ago (see Lisby et al, 2004) but has not been tested comprehensively as they did and thus is novel. Inclusion of biochemical, genetic and structural analyses further offer more complete picture of this process. I have a couple of questions and suggestions that require addressing experimentally and through rewriting the manuscript to clarify a few points.

1. Evidence suggests that removal of Mre11 complex from DNA ends could be coupled to resection event itself. For instance, sgs1-D664delta is deficient in resection and synthetic lethal to sae2 deletion, causing Mre11 to persist at DNA ends (Genetics. 2013 195(4):1241-51). The one model is thus that by virtue of endonuclease activity, Mre11 liberates itself from DNA and Sae2 could simply promote endonuclease activity of Mre11 to assist this process. In this scenario, one anticipates that mre11-H37R should bypass the requirement of Sae2 in endonucleolytic removal of Mre11 at DNA ends.

We agree with the reviewer that one key function of Sae2 is to promote Mre11 nuclease activity. However, our data suggest that Sae2 can also promote Mre11 removal from DSB ends in the absence of Mre11 nuclease activity, as is the case in the context of the *mre11-H125N* mutation. (Please also see discussion of point 2 of referee 1).

2. It is certainly logical that mre11-H37R could suppress sae2 deletion due to a reduced affinity to ssDNA and thereby becomes more prone to dissociate from DNA ends. Biochemical results support this idea. However, one cannot exclude the possibility that the mutation allows the endonuclease activity of other nuclease especially, Dna2, to gain access to and cleave DNA ends to substitute Mre11 endonuclease activity in sae2 deletion. The effect of DNA2 deletion on the CPT sensitivity of mre11-H37R sae2 should be tested.

We agree that, in the absence of Sae2 or Mre11, nuclease activities other endonucleases may take over the processing of the 5' end. This idea is in line with our new data (Figure E2B) in which we show that *mre11-H37R* suppresses the sensitivity of *sae2*Δ towards etoposide, where the 5' end is blocked by covalently bound Top2. However, similar to the synthetic lethality between *sae2*Δ and *sgs1*Δ, we could not recover a viable *dna2-1sae2*Δ strain to test this hypothesis.

3. Furthermore, resection in sae2 mre11-H37R exo1 should be examined in yeast strain following an induction of non-repairable HO break to test if the initial resection is improved by the mre11-H37R mutation.

We agree that it would be interesting to see the result of such experiment, but even if *mre11H37R* was able to improve the modest resection defect of a *sae2*Δ strain, this would unlikely be causative of the suppression. We note that there is little or no correlation between the extent/speed of resection as measured with the HO system and sensitivity to camptothecin: *sae2*Δ and *exo1*Δ strains show very similar weak defects in resection – 30 minutes delay at most (Clerici *et al*, 2006) – however, while the *sae2*Δ strain is extremely sensitive to camptothecin, the sensitivity of the *exo1*Δ strain is negligible (please see our work, figure 3G). Furthermore, despite both *sae2*Δ and *mre11*Δ strains showing strong hypersensitivity to DNA-damaging agents, the resection defect of the *sae2*Δ strain is much weaker than that of *mre11*Δ cells. In light of these points, we suggest that further analyses of resection rates of our various strains are unlikely to provide major new insights, and therefore request that such studies be considered beyond the scope of our current work.

4. In page 13, "mre11-nd cells are only impaired in Mre11 nuclease activity" I do not recall any studies testing the Mre11 level at DNA ends in mre11-H125N or tel1 mutants. It should be tested to

support this hypothesis.

While it is true that no one has tested if *mre11-H125N* is recruited to DNA ends by ChIP, a series of observations indicate that, apart from its lack of nuclease activity, Mre11-H125N is functional in other regards:

- Mre11-H125N is recruited to IR induced foci (Lisby *et al*, 2004).
- Cells expressing Mre11-H125N, unlike *mre11*Δ cells, are not defective in non-homologous end-joining, telomere maintenance or mating-type switching (Moreau *et al*, 1999).
- mre11-H125N forms a complex with Rad50/Xrs2 (Krogh *et al*, 2005).

We have mentioned these issues in our revised text (bottom of page 14).

5. The reference listed as "Chen et al" is without Journal information and could not be found from pubmed search.

We apologize for this mistake. We have learned from attending conferences that the laboratory of Dr. Lorraine Symington (New York, USA) has data that are very complementary to ours. Specifically, through targeted mutagenesis studies on Mre11 the Symington group identified some of the *mre11* mutations that we have identified in our study. Given that it was referring to an unpublished study, we have decided to remove this citation.

Referee #3:

This study by Puddu and Jackson et al. examines the role of the Sae2 protein in budding yeast and its relationship to the Mre11-Rad50-Xrs2 (MRX) complex in DNA repair. Sae2 null mutants are deficient in meiotic double-strand break repair and also show sensitivity to DNA damaging agents in mitotic cells. Here the authors select for genetic suppressors of the sae2 phenotype in yeast and identify a number of alleles in various genes that can nearly completely suppress the camptothecin sensitivity of a sae2 strain. Remarkably, one particular residue of the Mre11 protein is found to be the site of a missense mutation in several of these strains, and the authors show that this allele acts as a recessive suppressor of some of the mitotic phenotypes of sae2, but not its meiotic phenotype. In vitro analysis indicates that this mutant is not hyperactive for nuclease activity as one might expect, but appears to be partially deficient in DNA binding. This overall result coincides with a previous publication showing that sae2 strains retain the MRX complex for a longer time and at higher levels than a wild-type strain. In addition, the nearly complete suppression of sae2 camptothecin sensitivity by various alleles of Mre11 suggest that a significant component of the sae2 phenotype may be attributed to inappropriate MRX binding to DNA.

This is an important study that is likely to be of interest to many in the DNA damage response field. The data and presentation is generally sound, although I do not think there is sufficient data here to conclusively say what is the exact defect in Mre11 that is exhibited by the suppressor mutants. It could be a DNA-binding defect, although complete evidence for this is lacking. The model presented suggests that this is the foregone conclusion here, but better evidence for this as well as some other validation of the existing experiments is also necessary.

1. It is interesting that the H37Y/R suppressor mutations in Mre11 do not suppress the meiotic defect in sae2 strains. Since the protein-DNA conjugate in the case of Spo11 would be a 5' adduct, in contrast to the 3' TOP1 adduct with camptothecin, the authors should examine whether the mre11 suppressor allele works when sae2 strains are tested for survival on a topoisomerase II poison such as etoposide.

In response to these comments, we have introduced the *sae2*Δ and *mre11-H37R* mutations in an *erg6*Δ background, which allows greater permeability of the plasma membrane to etoposide. The results of our studies, provided in the new Figure E2B, show that the *mre11-H37R* mutation can suppress the hypersensitivity of *sae2*Δ cells to etoposide, suggesting somewhat different repair mechanisms for meiotic and etoposide-induced DSBs.

2. Since the H37 residue is highly conserved and is in a region predicted to contact DNA, as the authors point out, it seems unlikely that the H37A mutant is completely normal for DNA binding

while the H37R and H37Y mutants are not. This should be explicitly tested in vitro with purified proteins and in the Mre11 foci assay to determine if loss of binding affinity is actually the reason for the H37R/Y suppression of sae2 phenotypes.

As reported in our revised manuscript, we have carried out further biochemical studies focused on the Mre11 H37A mutant, which are now included in the figures 4C and 4D. As described in the text, this protein retains substantial ssDNA binding activity, further suggesting that it is the loss of this activity that is responsible for the suppression of *sae2*Δ DNA damage sensitivity.

3. The same issue also arises with the Q70R, L89V, and P110L mutants - are these all deficient in DNA binding in vitro? Do they show a specific defect in single-stranded DNA binding? Is there a preference for the polarity of single-stranded DNA binding? Also, the levels of these proteins in vivo should be confirmed as it was for the H37 mutants.

In our revised manuscript, we now provide a biochemical characterization of Mre11^{Q70R} which, as expected, behaves quite like Mre11^{H37R} in regard to single and double stranded DNA binding (new figure E3C). Additionally, we show that the *in vivo* levels of all Mre11 mutant proteins we have isolated are comparable to that of wild-type Mre11 (new figure E3B). We acknowledge that it will be interesting to explore the properties of the other various Mre11 mutant proteins in a range of assays. However, we point out that such studies are technically challenging and are likely to take a substantial amount of time, and that we have been informed by Prof. Lorraine Symington that a paper describing very complementary and substantially overlapping studies carried out in her laboratory has just been accepted for publication. In light of these issues, we request that such biochemical analyses are considered beyond the scope of our current work.

4. It is interesting that the sup28 and sup29 alleles of mre11 are recessive for suppression of the CPT phenotype of a sae2 strain (Fig. 2C). One would think that the same scenario is occurring in the presence of other types of damage, but it appears that the alleles are not recessive when measured for survival to MMS. Why is this?

We do not think that sup28 and sup29 are dominant for survival to MMS. The experiment in Figure 2C shows that expression of wild-type *MRE11* in the *sae2*Δ strains bearing the sup mutations, can sensitize cells to camptothecin and MMS (compare rows 3-4 with 7-8). However, combining the fact that the *sae2*Δ strain is less sensitive to MMS than to camptothecin at the doses used (compare the second rows in the camptothecin and MMS plates), and the fact that cells grow faster in the presence of MMS compared to camptothecin (compare the size of the wild-type colonies on the two plates) gives the impression that the mutation is not fully recessive. Whatever the case regarding these issues, we feel that the data support our conclusion that introduction of wild-type *MRE11* sensitized the *mre11*$^{SUPsae2\Delta}$ *sae2*Δ strains to DNA-damaging agents.

5. The message here seems to be that the sae2 damage sensitivity phenotype is primarily reflecting the block to DSB repair imposed by improperly bound MRX, and not from a deficiency in resection alone. If this is the case, why is SSA still impaired in the sae2 strain and is not recovered by mre11-H37R? Presumably this is not a resection defect? If so then what is the defect in a sae2 strain undergoing SSA?

The reviewer is correct: we think that the defect in SSA of a *sae2*Δ strain reflects a different function of Sae2. In this regard and as discussed in our revised manuscript (1/4 way down page 15), it has been previously suggested that Sae2 promotes SSA by it possessing a "bridging activity" that helps keep the two ends of a DSB in close proximity to each other, and two recently published biochemical studies with Sae2 counterparts from *S. pombe* and human (Ctp1 and CtIP, respectively) support this type of function (Andres *et al*, 2015; Davies *et al*, 2015). It seems logical that lack of such end-bridging in a *sae2*Δ strain would not be suppressed by reduced binding of Mre11 to DNA.

6. Fig. 4: Mre11 does not bind to DNA on its own - it is part of the larger complex with Rad50 and Xrs2. Are the H37R mutant and the other mutants forming the MRX complex normally in vivo? What is the DNA binding capacity of H37R in the form of MRX?

Given that both *rad50*Δ and *xrs2*Δ strains are extremely sensitive to DNA damage, and that, to our knowledge, mutations disrupting the formation of the MRX complex always result in phenotypes

comparable to the corresponding null alleles (DNA damage hypersensitivity and meiotic defects), we think it would be extremely unlikely if we could detect a suppression of DNA damage sensitivity if the formation of the MRX complex was impaired.

7. The fact that the H37R allele of Mre11 suppresses the camptothecin sensitivity of the Rad50S allele suggests that Rad50S versions of MRX may bind to single-stranded DNA with higher affinity or longevity compared to wild-type Rad50; is this the case?

Rad50 contains a DNA binding domain, which despite not being required for DNA repair, is important for telomere maintenance (Rojowska A, EMBO J, 2014). We did not test the ability of the M^{H37R}RX complex to bind DNA because we think that using the entire complex could lead to potential confounding effects due to these additional DNA binding sites.

8. It has been shown that in S.pombe, 5'-adduct removal depends on both Rad32(Mre11) and Ctp1(Sae2) while the removal of 3' adducts requires only Rad32. (http://www.ncbi.nlm.nih.gov/pubmed/19150433 [4]) It would be informative here if the authors could explain how the current study fits into these existing data.

We thank this reviewer for drawing our attention to this article, where the authors show that in *S. pombe* the camptothecin sensitivity of *mre11-nd* and *rad50S* alleles likely stems from lack of removal of covalently bound Topoisomerase 1 from DSB ends. We suggest that this defect may result from the stalling of the MRX complex on DNA ends, which could limit the accessibility of the DNA to other factors that are known to be important for the processing of Top1-DNA cleavage complexes such as Tdp1 or Rad1-Rad10. We included this possibility in our manuscript (2/3 down page 14). We cannot however explain the reason why *ctp1Δ* mutants are over-proficient in Top1 removal, but we note that there is a substantial difference between *S. pombe* Ctp1, which is required for 5' end resection and *S. cerevisiae* Sae2 which is not.

REFERENCES

Andres SN, Appel CD, Westmoreland JW, Williams JS, Nguyen Y, Robertson PD, Resnick MA & Williams RS (2015) Tetrameric Ctp1 coordinates DNA binding and DNA bridging in DNA double-strand-break repair. *Nat. Struct. Mol. Biol.* **22:** 158–66 Available at: http://www.ncbi.nlm.nih.gov/pubmed/25580577 [Accessed January 13, 2015]

Clerici M, Mantiero D, Lucchini G & Longhese MP (2006) The Saccharomyces cerevisiae Sae2 protein negatively regulates DNA damage checkpoint signalling. *EMBO Rep.* **7:** 212–8 Available at: http://www.pubmedcentral.nih.gov/articlerender.fcgi?artid=1369247&tool=pmcentrez&rendertype=abstract [Accessed October 7, 2011]

Davies OR, Forment J V, Sun M, Belotserkovskaya R, Coates J, Galanty Y, Demir M, Morton CR, Rzechorzek NJ, Jackson SP & Pellegrini L (2015) CtIP tetramer assembly is required for DNA-end resection and repair. *Nat. Struct. Mol. Biol.* **22:** 150–7 Available at: http://www.ncbi.nlm.nih.gov/pubmed/25558984 [Accessed January 5, 2015]

Krogh BO, Llorente B, Lam A & Symington LS (2005) Mutations in Mre11 phosphoesterase motif I that impair Saccharomyces cerevisiae Mre11-Rad50-Xrs2 complex stability in addition to nuclease activity. *Genetics* **171:** 1561–70 Available at: http://www.pubmedcentral.nih.gov/articlerender.fcgi?artid=1456084&tool=pmcentrez&rendertype=abstract [Accessed October 22, 2013]

Lisby M, Barlow JH, Burgess RC & Rothstein R (2004) Choreography of the DNA damage response: spatiotemporal relationships among checkpoint and repair proteins. *Cell* **118:** 699–713 Available at: http://www.ncbi.nlm.nih.gov/pubmed/15369670

Moreau S, Ferguson JR & Symington LS (1999) The nuclease activity of Mre11 is required for meiosis but not for mating type switching, end joining, or telomere maintenance. *Mol. Cell.*

Biol. **19:** 556–66 Available at:
http://www.pubmedcentral.nih.gov/articlerender.fcgi?artid=83913&tool=pmcentrez&rendertype=abstract [Accessed May 17, 2012]

Acceptance letter 02 April 2015

Thank you for submitting your final revised manuscript for our consideration. I am pleased to inform you that we have now accepted it for publication in The EMBO Journal.
Thank you again for this contribution to The EMBO Journal and congratulations on a successful publication! Please consider us again in the future for your most exciting work.

Referee #1:

The authors responded to my comments and I support the manuscript to be published in EMBO J

Referee #2:

The revision addressed most of my original comments and therefore becomes suitable for publication at EMBO Journal.

© European Molecular Biology Organization
